# Improving vaccination coverage and timeliness through periodic intensification of routine immunization: evidence from Mission Indradhanush

**DOI:** 10.1111/nyas.14657

**Published:** 2021-07-15

**Authors:** Amit Summan, Arindam Nandi, Sarang Deo, Ramanan Laxminarayan

**Affiliations:** ^1^ Center for Disease Dynamics Economics & Policy Washington DC; ^2^ Indian School of Business Hyderabad India; ^3^ Center for Disease Dynamics Economics & Policy New Delhi India; ^4^ High Meadows Environmental Institute Princeton University Princeton New Jersey

**Keywords:** India, vaccine, Mission Indradhanush, Universal Immunization Programme, UIP

## Abstract

Only an estimated 62% of Indian children under the age of 2 years are fully immunized. We examined the association between India's Mission Indradhanush (MI)—a periodic intensification of the routine immunization program—which was implemented in phases across districts between March 2015 and July 2017, and routine vaccination coverage and timeliness among children. We used data from a 2015 to 2016 national survey of children (*n* = 29,532) and employed difference‐in‐difference regressions to examine binary indicators of receipt of 11 vaccines and whether vaccines were received at recommended ages. The full immunization rate was 27% higher among children under 2 years old residing in MI phase 1 and 2 districts (intervention group) as compared with those residing elsewhere (control group). The rate of receiving all vaccines at recommended ages was 8% higher in the intervention group. Receiving doses of oral polio vaccine (OPV) birth dose, OPV dose 1 (OPV1), OPV2, OPV3, bacillus Calmette–Guérin, and hepatitis B birth dose vaccines were 9%, 9%, 11%, 16%, 5%, and 19% higher in the intervention group than the control group, respectively. More research is required on the cost‐effectiveness of investing in MI‐type programs as compared with routine immunization.

## Graphical abstract

Only an estimated 62% of Indian children under the age of two years are fully immunized. We examined the association between India's Mission Indradhanush (MI)—a periodic intensification of the routine immunization program—which was implemented in phases across districts between March 2015 and July 2017, and routine vaccination coverage and timeliness among children.

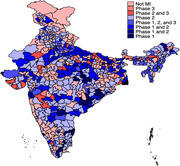

## Introduction

An estimated 400,000 Indian children under the age of 5 years die annually from vaccine‐preventable diseases, such as pneumonia, diarrhea, and measles.^1^ India's Universal Immunization Programme (UIP) is among the largest routine childhood immunization programs in the world. With an annual budget of $2 billion, the UIP aims to immunize 26 million newborn children every year.^2^, ^3^ The program currently provides the following vaccines: oral polio vaccine (OPV), diphtheria‐pertussis‐tetanus (DPT), bacillus Calmette–Guérin (BCG), measles, hepatitis B, *Haemophilus influenzae* type B (Hib) containing pentavalent (DPT, hepatitis B, and Hib), inactivated polio vaccine (IPV), tetanus toxoid, and in endemic areas, Japanese encephalitis. Rotavirus, pneumococcal, and measles‐rubella vaccines have also been introduced in select high‐burden areas.^4^

Despite the scale of the UIP, India has yet to achieve the universal coverage of routine childhood vaccines. In 2016, the most recent year for which national estimates are available, only 62% of 12‐ to 23‐month‐old Indian children received full immunization (BCG, measles, and three doses each of polio and DPT).^5^, ^6^, ^7^ In addition to low coverage, failure to vaccinate children at recommended ages has remained a major challenge. In 2013, the proportion of delayed doses among children under 5 years old ranged from 35% for OPV first dose (OPV1) to 65% for DPT3.^8^ Among 10‐ to 23‐month‐old children in 2016, the proportion of delayed doses (i.e., more than 28 days after the minimum eligibility age) ranged from 23% for BCG to 35% for the measles vaccine.^9^ Timely vaccination is important especially for highly contagious diseases such as measles that can rapidly affect a large number of children and hinder long‐term immunity against other diseases.^10^, ^11^

In December 2014, the Government of India launched Mission Indradhanush (MI), with the objective of increasing full immunization coverage.^12^ MI was a periodic intensification of the routine immunization (PIRI) program which targeted unvaccinated and undervaccinated children by allocating more resources to underserved areas. The program was implemented in 528 districts—with low initial full immunization coverage and high dropout rates—in four phases during April 2015–July 2017.^4^ An estimated 25.5 million children across India were vaccinated under MI in this time.^12^

Evidence on the impact of MI on desired programmatic and immunization outcomes is limited. Data from the Integrated Child Health and Immunization Survey (INCHIS) in 24 states showed an increase in full immunization rates from 64.1% to 73.5% during April 2015–October 2015.^13^ In districts which were covered under phase 1, rates of OPV third dose and DPT third dose increased substantially. However, these estimates did not control for potential confounding factors and secular time trends of vaccination coverage in MI and non‐MI areas. A study found that the Intensified Mission Indradhanush (IMI) program—a successor of MI—may have increased coverage rates by 3.9–35.7% for different vaccines; however, this study inferred coverage rates through vaccine delivery volume rather than data on administration of vaccine to individual children.^14^

We estimated the association between exposure to MI phases 1 and 2, and child immunization outcomes by comparing vaccination rates (overall, as well as at recommended ages) and timing in MI vis‐à‐vis non‐MI districts. We used household survey data and employed difference‐in‐difference (DID) multivariate regression models that controlled for several possible confounders.

## Data and methods

### INCHIS survey data

We used data from the Integrated Child Health and Immunization Survey (INCHIS), a stratified, nationally representative, cross‐sectional household survey conducted in three rounds from March 2015 to April 2016.^13^ The survey covered a total of 44,571 households across 260 districts in 24 states across the three rounds. Data were obtained on socioeconomic and demographic indicators, and access to and quality of health facilities for households with children below the age of 2 years. Immunization receipt and date information for the youngest child under 2 years old in each household were collected from either the vaccination card or through maternal (or caregiver) recall if the card was unavailable.

Each round of INCHIS collected data from 12 states, where Bihar, Maharashtra, Madhya Pradesh, Rajasthan, Telangana, and Uttar Pradesh were fixed, and the six other states were rotated across rounds. The states were chosen to ensure representation from each geographical region and level of development. Within each state, a three‐stage stratified sampling design was employed where district, cluster (village/urban ward), and households were selected at three different stages. Districts were stratified into three or four groups based on a composite index using the following district‐level indicators: proportion of urban households, percentage of scheduled caste/tribe population, literacy rate, proportion of households with latrine facility, and availability of banking facilities. Within each stratum, 1–3 districts were chosen based on the population of the state. Within a selected district, systematic sampling was used to draw clusters where the sampling frame (2011 census data) of clusters was ordered by female literacy rate. Probability sampling was used to select the household, where households with at least one child between the ages of 0 and 23 months were eligible for selection within the cluster.^13^

**Figure 1:**
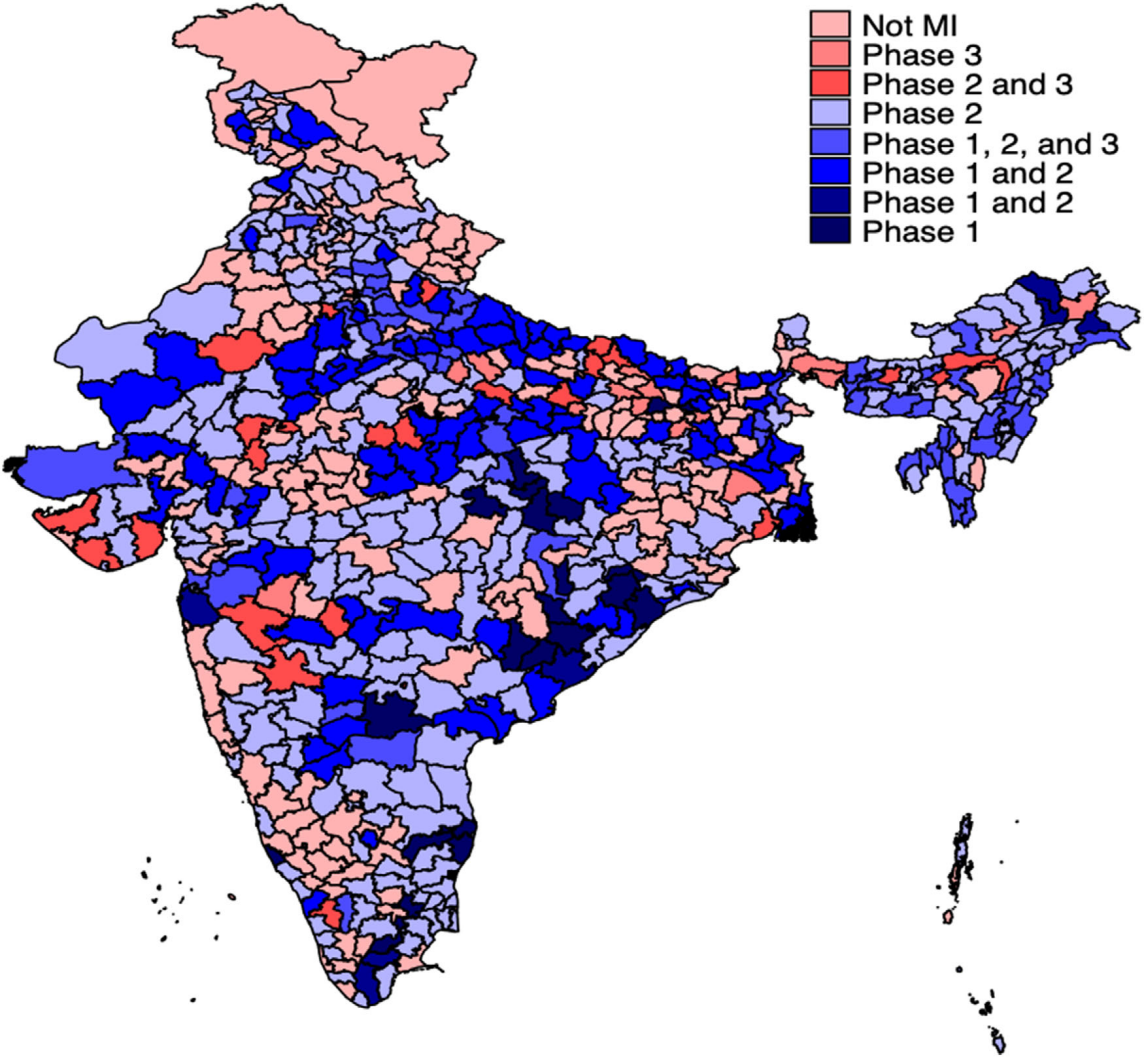
District‐wise implementation of Mission Indradhanush phases 1, 2, and 3. Map coordinate data are from the Database of Global Administrative Areas, version 2.8 (2015), combined with district identifiers from the National Family and Health Survey of India 2015–2016. Colors denote the phases of Mission Indradhanush (MI) implemented in each district. Districts with no data are colored white. This map is for illustration only and may not depict correct international boundaries.

Initially, MI was rolled out across India in two phases. Some districts were included in the program during both phases 1 and 2, while others were included only in either phase. We analyzed the impact of the first two phases of the MI program (April–July 2015, and October 2015–January 2016, respectively) using data from the first and last rounds of INCHIS. INCHIS‐1 was conducted before the MI program, INCHIS‐2 was conducted in between MI phases 1 and 2, and INCHIS‐3 was conducted after the conclusion of MI phase 2. MI phases 1 and 2 covered 480 districts of India, 77 of which were included in the INCHIS‐1 and INCHIS‐3 rounds. Figure 1 shows the MI phases across districts in a map of India, and Figure S1 (online only) shows the timing of INCHIS rounds and MI phases.

In our study, children from districts included in phases 1 and 2 (P1&2) of MI were considered as the intervention group. The control group included children from districts that were not covered in either MI phase. Children in INCHIS‐1 were considered preintervention observations, while children in INCHIS‐3 were postintervention observations. There were 48 P1&2 districts eligible for analysis—21 MI districts: 13 before and 8 after the intervention; and 27 non‐MI districts: 8 surveyed before and 19 after the intervention. This resulted in data on 9674 children available for analysis, 3929 in the MI group and 5745 in the non‐MI group.

### Analysis

We explored the association of MI (phases 1 and 2) and the binary indicators of 11 vaccination outcomes for each child: full immunization, receipt of diphtheria‐pertussis‐tetanus dose 1 (DPT1), DPT2, DPT3, oral polio vaccine dose 1 (OPV1), OPV2, OPV3, OPV birth dose (OPV0), measles first dose (measles1), bacillus Calmette–Guérin (BCG), hepatitis B birth dose (HepB0), and on‐time vaccination (OTV). The IPV was introduced into the UIP in 2016 after MI phase 3 and is, therefore, not included in our analysis. The eligibility age for each vaccine was taken from the Indian Academy of Pediatrics and World Health Organization guidelines.^15^, ^16^ Table S1 (online only) describes each vaccine and its earliest eligibility age for a child. Each child who had reached the vaccination eligibility age before the end of the MI intervention was considered for analysis for that vaccine. We excluded children who were reported as vaccinated before the eligibility age (0.6% of the sample) to reduce potential measurement errors.

The OTV indicator considered the timely receipt of DPT1, DPT2, DPT3, and full immunization (three doses each of DPT and OPV, and one dose each of measles and BCG). Each child was evaluated for timely vaccination of the latest vaccine dose they were eligible for, resulting in one observation per child for OTV. The indicator had a value of 1 if the child had received the dose within 28 days after the earliest age of eligibility described in Table S1 (online only). Previous studies had considered vaccination as timely if done within 28–30 days after eligibility.^17^, ^18^

We employed a DID framework in which the average difference in outcomes before and after the MI program in each district was first estimated. Then, the difference between MI and control districts of this average difference was taken. Each model included binary indicators of location (whether an MI district), time (pre‐ or postintervention), and an interaction between the two (DID indicator). We used linear probability models (LPM) and probit models for our analysis.

Our regression models included the following household‐level socioeconomic indicators and mother and child characteristics as covariates: locality (urban), caste (scheduled caste, scheduled tribe, and other backward classes), religion (Muslim, Christian, Sikh, and other religions), household size, wealth quintiles, age of the mother, mother's schooling attainment (primary or lower, middle to secondary, and graduate and above), child's age, sex, and place of birth (health facility or home). We also included the distance to the nearest public health subcenter (SC). The SC is the first point of contact between the primary health system and patients and is used as a measure of accessibility to health services.^19^ Finally, we used principal component analysis to create a wealth index of physical household characteristics and asset ownership. We included indicators of the top four wealth index quintiles in the model, keeping the bottom quintile as the reference category. A detailed description of the construction of the wealth index is provided in the Supplementary Text (online only). All covariates were obtained from the INCHIS data. Standard errors were clustered at the district level and survey weights were applied. Data were analyzed using STATA version 14.2 and we considered *P* < 0.05 to be statistically significant.

### Parallel trends test

The parallel trends test is an important measure of methodological validity in DID analyses. If the parallel trends test were to be satisfied, child vaccination status in MI and non‐MI districts should follow a similar trend in the years leading up to MI introduction. We used data from the National Family Health Survey 2015–2016 (NFHS‐4) to test if trends in vaccination status in MI and non‐MI districts were statistically indistinguishable between 2011 and 2014. NFHS‐4 is a nationally representative survey that collected the vaccination history information of 259,627 Indian children under 5 years old. Data were taken from the vaccination card or the caregiver when the vaccination card was unavailable. We compared DPT3 child vaccination rate trends between intervention and control districts. DPT3 is a widely used indicator for vaccination coverage, timely vaccination, and the overall performance of the system. For children who did not have a vaccination date recorded, we assumed that the child was vaccinated at the DPT3 eligibility age (14 weeks since birth).

We tested for parallel trends in two ways.^20^ First, we estimated district fixed effect regression models of DPT3 vaccination rates for children from 2011 to 2014. We examined if the time trends of the estimated residual error terms of these models were parallel across MI and non‐MI districts. Second, we regressed the rate of DPT3 vaccination on year identifiers, binary indicator of whether a district was included in MI, and interaction terms of the year and MI identifiers. If the estimated regression coefficients of the interaction terms were not statistically significant, the parallel trends assumption would be satisfied, that is, trends in DPT3 vaccination rates would be similar between MI and non‐MI districts leading up to the implementation of MI.

## Results

### Summary statistics of the study sample

**Figure 2:**
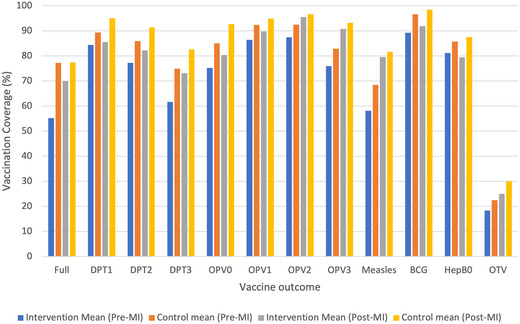
Vaccination outcomes by control and intervention groups before and after MI implementation. BCG, bacillus Calmette–Guérin; DPT, diphtheria‐pertussis‐tetanus; Full, one dose BCG and measles, three doses of DPT and polio; HepB0, hepatitis B given at birth; Measles1, first dose of measles; OPV, oral polio vaccine; OPV0, OPV birth dose; OTV, on‐time vaccination—considers timely vaccination of DPT and full immunization.

Table S2 (online only) shows the differences in vaccination rates and socioeconomic and demographic characteristics of children across the intervention and control groups for the baseline period. Vaccination rates were higher in the control group for all 11 vaccines. The largest unadjusted differences in vaccination rates between P1&2 intervention and control groups were for full immunization (55% versus 77%, *P* < 0.01), measles1 (75% versus 85%, *P* < 0.01), and DPT3 (62% versus 75%, *P* < 0.01). Figure 2 shows vaccination outcomes between control and intervention groups before and after the MI program. The unadjusted gap between MI and non‐MI districts reduced substantially from the pre‐ to post‐MI period for most vaccination outcomes, except for slight increases for DPT1, DPT2, OPV0, HepB0, and OTV. Among covariates, the largest unadjusted baseline differences were for children who were not born at health facilities (31% in MI versus 15% in non‐MI districts, *P* < 0.01) and proportion of minority religion households (11% in MI versus 38% in non‐MI districts, *P* < 0.01).

Table S3 (online only) shows the distribution of children by immunization status and socioeconomic characteristics. Rates of vaccination were higher among children in richer and urban households, Sikh and general caste households, those born in health facilities, and those with more educated mothers, as compared with the respective reference groups. Among the outcome indicators, the differences across socioeconomic groups were the largest for full immunization and OTV rates.

### Parallel trends test results

**Figure 3:**
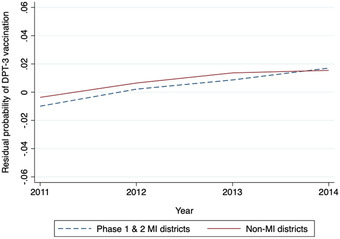
Average annual residual probability of DPT vaccination in MI and non‐MI districts, 2011–2014. *n = *178 districts: 126 non‐MI and 52 MI districts. DPT, diphtheria‐pertussis‐tetanus. Time trends of the estimated residual error terms of district fixed‐effects regression of DPT3 vaccination rates on year from 2011 to 2014.

**Figure 4:**
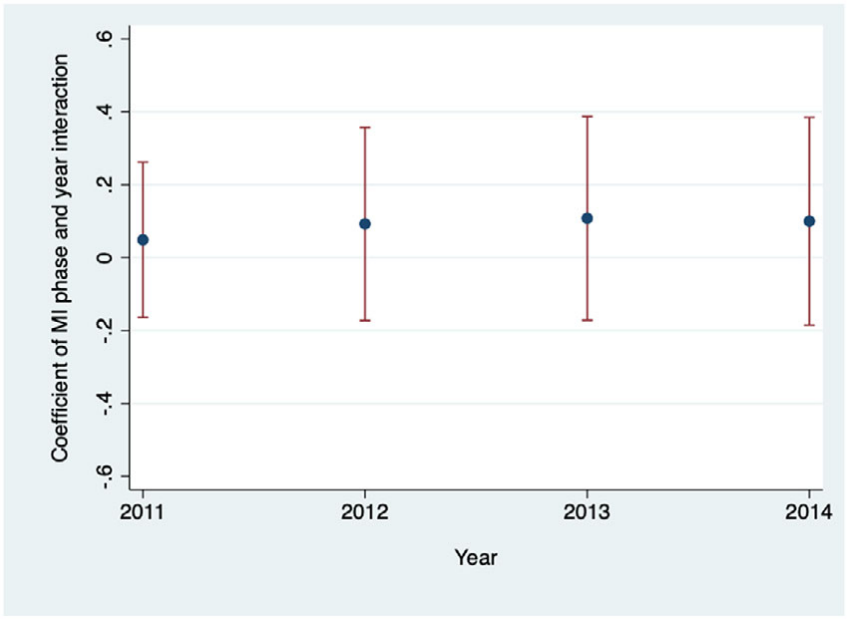
Coefficient of interaction between MI districts and year in the regression of DPT vaccination, 2011–2014. *n* = 178 districts: 126 non‐MI and 52 MI districts. DPT, diphtheria‐pertussis‐tetanus. Coefficient of MI phase year interaction term with 95% confidence interval from district fixed‐effects regression of the rate of DPT3 vaccination on year identifiers, the binary indicator of whether a district was included in MI, and interaction terms of the year and MI identifiers.

Figure 3 shows the residual probability of the DPT3 vaccination rate between MI P1&2 districts and non‐MI districts from 2011 to 2014. Time trends of the residual DPT3 vaccination probability were similar for MI and non‐MI districts. Figure 4 presents the estimated coefficients (with 95% confidence intervals) of the interaction terms between year and MI indicators in the regression of DPT3 vaccination rate. Leading up to the introduction of MI in 2015, there was no statistical difference between DTP3 vaccination rates in the intervention and control districts. Therefore, the parallel trends assumption was satisfied.

### Regression results

Table 1 presents summary results of the DID analysis, showing only the estimated coefficients of the DID indicator for both the LPM and probit model. The DID likelihood of receiving full immunization was 27% (95% confidence interval [CI]: 0.11–0.42, *P* < 0.01, LPM) higher among children under 2 years old residing in MI phase 1 and 2 districts (intervention group) as compared with those residing elsewhere (control group).

**Table 1:** Summary of linear probability regression results for immunization outcomes and Mission Indradhanush phase 1 and 2

		Linear probability model		Probit model		
Model	Vaccine	Coefficient	*R* ^2^	Coefficient	Pseudo *R* ^2^	*n*
1	Full	0.27**	0.155	0.26**	0.155	4474
		0.08		0.08		
2	DPT1	0.02	0.107	0.02	0.107	8603
		0.03		0.03		
3	DPT2	0.07^+^	0.133	0.06	0.133	8272
		0.04		0.03		
4	DPT3	0.15^+^	0.164	0.14	0.164	7917
		0.08		0.08		
5	OPV0	0.09*	0.131	0.07*	0.131	9033
		0.03		0.03		
6	OPV1	0.09**	0.126	0.06*	0.126	8699
		0.02		0.02		
7	OPV2	0.11*	0.183	0.09*	0.183	8501
		0.04		0.04		
8	OPV3	0.16**	0.219	0.15**	0.219	8282
		0.06		0.05		
9	Measles1	0.05	0.129	0.04	0.129	5651
		0.05		0.05		
10	BCG	0.05*	0.1	0.04	0.1	9033
		0.02		0.02		
11	HepB0	0.19**	0.167	0.16**	0.167	9033
		0.04		0.04		
12	OTV	0.08*	0.178	0.09*	0.178	8315
		0.04		0.04		

Note: Includes district‐level fixed effects. Probit model shows average marginal effects. Standard errors are shown below the coefficients. BCG, bacillus Calmette–Guérin; DPT, diphtheria‐pertussis‐tetanus; Full, one dose BCG and measles, three doses of DPT and polio; HepB0, hepatitis B given at birth; Measles1, first dose of measles; OPV, oral polio vaccine; OPV0, OPV birth dose; OTV, on‐time vaccination—considers timely vaccination of DPT and full immunization. ^+^ *P* < 0.1; ^*^ *P* < 0.05; ^**^ *P* < 0.01.

Table S4 (online only) presents the full LPM results. The DID likelihood of children in the P1&2 intervention groups was also 9% higher for OPV0 (CI: 0.02–0.15, *P* < 0.05, LPM), 9% higher for OPV1 (CI: 0.04–0.14, *P* < 0.01, LPM), 11% higher for OPV2 (CI: 0.02–0.19, *P* < 0.05, LPM), 16% higher for OPV3 (CI: 0.04–0.27, *P* < 0.01, LPM), 5% higher for BCG (CI: 0.01–0.09, *P* < 0.05, LPM), and 19% higher for hepatitis B birth dose (CI: 0.11–0.28, *P* < 0.01, LPM). The DID likelihood in the P1&2 intervention group to have received age‐appropriate vaccines as per recommended schedule (OTV) was 8% higher (CI: 0.00–0.15, *P* < 0.05, LPM) than the control group.

The coefficients of the DID indicator in the probit model were almost identical to those in the LPM. The DID likelihood of children in the P1&2 intervention groups was 26% higher for full immunization (CI: 0.12–0.34, *P* < 0.01), 7% higher for OPV0 (CI: 0.00–0.26, *P* < 0.05), 6% higher for OPV1 (CI: 0.04–0.14, *P* < 0.05), 9% higher for OPV2 (CI: 0.02–0.19, *P* < 0.05), 15% higher for OPV3 (CI: 0.04–0.27, *P* < 0.01), 16% higher for hepatitis B birth dose (CI: 0.01–0.37, *P* < 0.01), and 9% higher for OTV (CI: 0.03–0.13, *P* < 0.05) than children in the control group. However, the results were not significant for measles1 in the probit model. Figures S2–S13 (online only) show the distribution of coefficients and significance levels for the estimated DID indicators from the probit models. For full immunization, hepatitis B birth dose, and OTV, the DID indicator coefficients are significant for all observations. In additional analysis, we found that vaccination outcomes were not statistically different in districts that were covered by either MI phase 1 or phase 2, but not both, as compared with non‐MI control districts (Tables S5 and S6, online only).

The covariates indicated that children in higher wealth quintiles were more likely to be vaccinated compared with the lowest wealth quintile. The magnitude of the estimated association increased with the household's wealth quintile. Maternal schooling level was positively associated with vaccination for all vaccines except for DPT3 and measles1, and institutional delivery was positively associated with vaccination outcomes across all vaccines.

## Discussion

In 2018, there were 882,000 deaths among Indian children under the age of 5 years;^21^ and an estimated 500,000 of these deaths could have been prevented with childhood vaccines.^1^ Significant disparities in mortality rates across states—in 2016, mortality rates for children under 5 years old varied from 65 per 1000 live births in Madhya Pradesh to 7 per 1000 live births in Kerala^22^—mirror the vaccination disparities across states. Only 62% of Indian children under the age of 23 months were fully vaccinated in 2016.^22^ Furthermore, of those who were vaccinated, vaccination was often delayed—only 35% of Indian children in 2012 reported receiving DPT3 vaccination within a month of the recommended age,^8^ and only 23% of children received BCG vaccination within 28 days of the recommended age during 2015–2016.^17^

Vaccination timing is especially critical for reducing the burden of highly infectious diseases. A child infected with measles can infect another 12–18 individuals, whereas someone with polio can infect another 5–7 individuals.^23^, ^24^ Timely vaccination can provide protection at the individual level and stop rapid outbreaks of these diseases through secondary protection. A study from 45 low‐ and middle‐income countries (LMICs) found large variations in timely vaccination and estimated that in approximately 25% of countries children were vaccinated close to their vaccination schedule.^25^ However, analysis of vaccination timeliness may be difficult in LMICs due to lack of good quality data on vaccination dates.

Our findings suggest that PIRI activities, through programs, such as MI, could play an important role in improving vaccination outcomes. We found that children under 2 years old in MI districts (those under both phases 1 and 2) had higher rates of full immunization, OPV0, OPV1, OPV2, OPV3, hepatitis B birth dose, BCG, and OTV as compared with children residing in non‐MI districts. We found that vaccination outcomes were not statistically different in districts which were covered by either MI phase 1 or phase 2, but not both, as compared with non‐MI control districts. This indicates that program duration may be a key factor for success.

Extensions of MI and other future PIRI activities can potentially follow the success of the Pulse Polio program.^26^, ^27^ The pulse polio immunization program included supplementary immunization sessions that achieved success through engagement with local stakeholders and effective tracking of beneficiaries.^26^ In 1995, the program was introduced nationwide, providing polio vaccines to 88 million children under the age of 3 years, and eventually covering all children under 5 years old.^27^ Between 1999 and 2018, the coverage of the polio vaccine third dose among Indian children 12–23 months old increased from 57% to 89%,^8^ and India was declared polio‐free in 2011.^28^

While our results suggest that PIRI activities can be successful, their long‐term effectiveness and financial viability are unclear.^27^ MI was succeeded by IMI, with a goal of reaching 90% full immunization coverage in the poorest performing districts by December 2018,^4^, ^12^ but it is unknown if this target was attained. A survey study showed an increase in full immunization rates in IMI districts.^12^ Another new study used administrative data on vaccine doses delivered and found increased delivery of doses to IMI districts during the intervention period for 13 vaccines, but reduced volume of vaccine delivery at the end of IMI.^14^ This suggests that further research is needed to evaluate the longer‐term effects of PIRI activities. While the IMI study used data on doses delivered to districts, child‐level data on vaccinations received before, during, and after a PIRI program may be more suitable for evaluating the program's effectiveness. The cost of the PIRI program per dose delivered needs to be compared to the routine immunization cost to evaluate its cost‐effectiveness. Future evaluations of MI should consider this.

PIRI activities can compensate for the inability of UIP to reach underserved communities but may not be a long‐term replacement for routine immunization. A health systems approach for improving routine immunization coverage and timeliness requires focus on the following four areas: financing, service delivery, stewardship, and creating and maintaining human resources.^28^

Adequate financial resources must be provided to UIP to improve long‐term vaccination outcomes. Although immunization budgets have increased in response to the introduction of new vaccines in the UIP, they have not kept pace with resource requirements—UIP suffered from estimated annual budgetary shortfalls of $9–$544 million during 2013–2017,^3^ which may be responsible for current gaps in vaccination coverage. The UIP budget is projected to increase by 41% from 2018 to 2022,^29^ but this is primarily to facilitate the introduction or universalization of new vaccines (e.g., pneumococcal)^30^ and to compensate for reductions in funding from Gavi, the Vaccine Alliance,^31^—which pays for 3% of India's immunization program budget at present.^3^

Resource allocation for immunization programs, including PIRI activities, should focus on fixing key supply‐side gaps in the UIP that affect service delivery.^32^ Post‐MI surveys suggest that inadequate and poor‐quality infrastructure and lack of human resources are among the most important supply‐side factors leading to lower participation in MI sessions.^12^ These factors have also slowed the rollout of newer vaccines, such as the hepatitis B vaccine.^33^ It will be important to integrate overall best practices in record keeping and vaccine management from high‐performing districts to poorer performing districts, in addition to providing additional investments in vaccine delivery systems and infrastructure in these districts.

India's shortage of a skilled health workforce affects child immunization, stretching community‐level health workers who serve as shared personnel for multiple tasks.^32^, ^34^ Greater allocation of staff resources toward routine immunization activities, including regularly updating immunization records, should be a priority.^12^ A recent study of MI from western India showed that 10% of study sites did not have an updated list of beneficiaries (known as the “due list”), 13% of auxiliary nurse midwives did not give all key messages about immunization, and 17% of community health workers were not aware about the incentive pay structure under the MI program.^35^ Investments in training programs with a focus on these particular tasks can help improve service delivery.

Strong governance and political commitment are critical for universal routine childhood vaccine coverage.^29^, ^36^, ^37^ To secure financing, build and retain skilled public health workers, and improve vaccination coverage, there must be a robust policy decision‐making process, sensible regulation, and a high level of health intelligence.^28^ Evidence‐based policymaking should be aided by efficient surveillance system akin to the highly successful acute flaccid paralysis surveillance.^28^ Greater power devolution to the National Technical Advisory Group on Immunization can expedite the policy‐making process on urgent matters, such as the introduction of new vaccines.^28^

Finally, it is important to consider the demand‐side drivers of vaccination to improve program success. Post‐MI surveys revealed the main demand‐side factors contributing to families not attending immunization sessions were lack of awareness, concerns about adverse effects of vaccines, and lack of time caused by mother and child illness.^12^, ^38^ To address these barriers, information campaigns should be used to promote the life‐saving benefits of vaccines, as well as their secondary and longer‐term positive effects on overall health, cognitive and educational outcomes, and reductions in potential medical expenditure.^11^, ^39^, ^40^, ^41^, ^42^, ^43^, ^44^ A recent systematic review of interventions to improve immunization coverage in LMICs found health education through home or village‐level meetings to be successful.^38^ Our results and findings from previous studies showed maternal schooling to be significantly positively associated with child vaccination rates.^45^ Therefore, health education interventions should target parents with lower levels of education.

Our analysis has important limitations. Our estimated associations of MI with vaccination outcomes may not be causal in the presence of unobserved covariates. For example, parents may decide to vaccinate their children based on perceived likelihood of survival of nutritional status, or they may be influenced by health workers to vaccinate certain children at higher rates. If such factors are correlated with MI status (e.g., frequent contact with health workers in MI districts), our estimated associations may be biased. However, we included a wide range of covariates in our regression models that are important determinants of vaccination outcomes and that have been used commonly in this type of analysis.

In INCHIS‐1 and INCHIS‐3, 40% and 34%, respectively, of households did not have a vaccination card available or did not show vaccination card to the surveyor, and the vaccination outcomes of the child were reported by the mother or caregiver. These observations can be susceptible to measurement errors if there were systematic differences in vaccination status of missing observations between intervention and control districts. We conducted additional analysis by only including households that had vaccination card available and where verification was completed by the surveyor. The results were similar to the main results (Table S7, online only).

Finally, INCHIS did not survey the same districts in multiple rounds. Therefore, our analysis could not compare changes over time in the same intervention and control districts, although we did control for confounding variables. Analysis of later MI phases can investigate richer datasets that do not have this limitation, more specifically following the same households over time would provide the most robust estimates of MI program effects.

Our study shows that large‐scale PIRI activities, such as MI, can potentially improve vaccination coverage and OTV rates in India. Further research using longer‐term data may provide more robust estimates of the potential effects of the program. Research on the cost‐effectiveness of PIRI programs could help inform resource allocation for routine immunization vis‐à‐vis supplementary programs, such as MI, in the long term.

## Author contributions

A.S., A.N., and R.L. designed the study. A.S. conducted the analysis and A.S. and A.N. wrote the first version of the manuscript. All authors interpreted the findings and critically evaluated and edited the manuscript. All authors approved the final draft for publication.

## Competing interests

The authors declare no competing interests.

## Supporting information


**Construction of Wealth Index**.
**Table S1**. Description of vaccination indicators.
**Table S2**. Distribution of households by outcome variables and socioeconomic and demographic characteristics for P1&2 intervention and control groups before intervention (%).
**Table S3**. Background characteristics of study children by vaccination status (%).
**Table S4**. Immunization outcomes and phase 1 and 2 Mission Indradhanush treatment.
**Table S5**. Immunization outcomes and phase 1 Mission Indradhanush treatment.
**Table S6**. Immunization outcomes and phase 2 Mission Indradhanush treatment.
**Table S7**. Immunization outcomes and phase 1 and 2 Mission Indradhanush treatment, vaccination card seen
**Figure S1**. Timeline of MI and INCHIS phases.
**Figure S2**. Full immunization interaction effects.
**Figure S3**. DPT1 interaction effects.
**Figure S4**. DPT2 interaction effects.
**Figure S5**. DPT3 interaction effects.
**Figure S6**. OPV0 interaction effects.
**Figure S7**. OPV1 interaction effects.
**Figure S8**. OPV2 interaction effects.
**Figure S9**. OPV3 interaction effects.
**Figure S10**. Measles interaction effects.
**Figure S11**. BCG interaction effects.
**Figure S12**. Hepatitis B birth dose interaction effects.
**Figure S13**. OTV interaction effects.
