# Supplementary material for: Improving vaccination coverage and timeliness through periodic intensification of routine immunization: evidence from Mission Indradhanush
Source: Ann N Y Acad Sci. 2021 Jul 15;1502(1):110–20. doi: 10.1111/nyas.14657 (PMC9291841; doi:10.1111/nyas.14657)
Supplement: Supplementary file 1 — Construction of Wealth Index. Table S1. Description of vaccination indicators. Table S2. Distribution of households by outcome variables and socioeconomic and demographic characteristics for P1&2 intervention and control groups before intervention (%). Table S3. Background characteristics of study children by vaccination status (%). Table S4. Immunization outcomes and phase 1 and 2 Mission Indradhanush treatment. Table S5. Immunization outcomes and phase 1 Mission Indradhanush treatment. Table S6. Immunization outcomes and phase 2 Mission Indradhanush treatment. Table S7. Immunization outcomes and phase 1 and 2 Mission Indradhanush treatment, vaccination card seen Figure S1. Timeline of MI and INCHIS phases. Figure S2. Full immunization interaction effects. Figure S3. DPT1 interaction effects. Figure S4. DPT2 interaction effects. Figure S5. DPT3 interaction effects. Figure S6. OPV0 interaction effects. Figure S7. OPV1 interaction effects. Figure S8. OPV2 interaction effects. Figure S9. OPV3 interaction effects. Figure S10. Measles interaction effects. Figure S11. BCG interaction effects. Figure S12. Hepatitis B birth dose interaction effects. Figure S13. OTV interaction effects. [file NYAS-1502-110-s001.docx]

**Supplementary Online Appendix**

**Construction of Wealth Index**

A wealth index is a composite measure of a household’s economic status and commonly used as a proxy of the long-term economic well-being of a household.^1^ Principal component analysis (PCA) was used to construct the wealth index. PCA is widely used method to reduce dimensionality through orthogonal linear transformation of the underlying data.^2^ The orthogonal unit eigenvectors calculated from a covariance matrix of data are known as the principal components in a multivariate setting. The following variables from the Integrated Child Health Immunization Survey (INCHIS) were used to construct the PCA: type of household structure; number of persons sharing a room for sleeping; availability of separate room used for kitchen, type of cooking fuel used; availability of electricity; toilet facility type; and ownership of clock, television, laptop, internet, refrigerator, washing machine, air cooler/air conditioner, motorcycle/scooter and car/jeep/van/tractor. We considered the first principal component, which captures most of the variance in data, as an index of wealth. The index was then divided into quintiles

Table S1: Description of vaccination indicators

| Vaccine | Definition | Recommended age |
| --- | --- | --- |
| Fully vaccinated | A child is considered fully immunized when they receive one dose of Bacillus Calmette–Guérin, three doses of DPT and polio and one dose of measles. Full immunization can occur in children as early as 12 months of age. | 12 months |
| DPT1 | Provides vaccination against diphtheria, pertussis (whooping cough), and tetanus, and requires three doses and a fourth booster dose. | 6 weeks |
| DPT2 |  | 10 weeks |
| DPT3 |  | 14 weeks |
| OPV0 | Provides vaccination against [poliomyelitis](https://en.wikipedia.org/wiki/Poliomyelitis) (polio). Requires one birth dose and three follow-up doses. As of 2018, OPV has been replaced by inactivated polio vaccine (IPV) in the immunization schedule. | at birth |
| OPV1 |  | 6 weeks |
| OPV2 |  | 10 weeks |
| OPV3 |  | 14 weeks |
| Hep B0 | Hepatitis B | at birth |
| Measles1 | First dose of measles vaccine | 9 months |
| BCG | Bacillus Calmette–Guérin | at birth |
| OTV | The on-time vaccination indicator considered the timely receipt of DPT1, DPT2, DPT3, and full immunization. Each child was evaluated for timely vaccination of the most recent vaccine they were eligible for. The indicator had a value of 1 if the child had received one of these vaccines within 28 days after the earliest age of eligibility. | See above |

Source: Indian Academy of Pediatrics^3^

**Table S2: Distribution of households by outcome variables and socioeconomic and demographic characteristics for P1&2 intervention and control groups before intervention (%)**

|  | **Intervention Mean** | **Intervention SD** | **Control mean** | **Control SD** | **Difference** |
| --- | --- | --- | --- | --- | --- |
| **Vaccine** |  |  |  |  |  |
| Full | 0.553 | 0.498 | 0.773 | 0.419 | -0.22** |
| DPT1 | 0.845 | 0.362 | 0.894 | 0.309 | -0.049** |
| DPT2 | 0.773 | 0.419 | 0.859 | 0.348 | -0.086** |
| DPT3 | 0.617 | 0.486 | 0.750 | 0.433 | -0.133** |
| OPV0 | 0.752 | 0.432 | 0.850 | 0.357 | -0.098** |
| OPV1 | 0.865 | 0.342 | 0.924 | 0.265 | -0.06** |
| OPV2 | 0.874 | 0.332 | 0.925 | 0.263 | -0.051** |
| OPV3 | 0.760 | 0.427 | 0.829 | 0.376 | -0.069** |
| Measles | 0.582 | 0.493 | 0.684 | 0.465 | -0.103** |
| BCG | 0.893 | 0.310 | 0.967 | 0.180 | -0.074** |
| HepB0 | 0.812 | 0.391 | 0.858 | 0.350 | -0.045** |
| OTV | 0.183 | 0.387 | 0.225 | 0.418 | -0.042** |
| **Sex** |  |  |  |  |  |
| Female | 0.487 | 0.500 | 0.466 | 0.499 | 0.020 |
| **Locality** |  |  |  |  |  |
| Urban | 0.344 | 0.475 | 0.298 | 0.457 | 0.046** |
| **Wealth Quintile** |  |  |  |  |  |
| 1 | 0.166 | 0.372 | 0.143 | 0.350 | 0.024* |
| 2 | 0.186 | 0.389 | 0.193 | 0.394 | -0.007 |
| 3 | 0.160 | 0.367 | 0.259 | 0.438 | -0.099** |
| 4 | 0.220 | 0.414 | 0.224 | 0.417 | -0.004 |
| 5 | 0.265 | 0.441 | 0.179 | 0.383 | 0.086** |
| **Religion** |  |  |  |  |  |
| Hindu | 0.618 | 0.486 | 0.889 | 0.314 | -0.271** |
| Muslim | 0.138 | 0.345 | 0.057 | 0.231 | 0.081** |
| Christian | 0.235 | 0.424 | 0.009 | 0.095 | 0.226** |
| Sikh | 0.006 | 0.080 | 0.039 | 0.193 | -0.032** |
| Other | 0.000 | 0.000 | 0.004 | 0.060 | -0.004** |
| **Caste** |  |  |  |  |  |
| General | 0.217 | 0.412 | 0.274 | 0.446 | -0.057** |
| Scheduled Tribe | 0.260 | 0.439 | 0.054 | 0.227 | 0.206** |
| Other Backward Caste | 0.386 | 0.487 | 0.306 | 0.461 | 0.08** |
| Scheduled Caste | 0.134 | 0.341 | 0.363 | 0.481 | -0.229** |
| **Education** |  |  |  |  |  |
| No Schooling | 0.262 | 0.440 | 0.234 | 0.423 | 0.029* |
| Primary Or Lower | 0.211 | 0.408 | 0.148 | 0.355 | 0.063** |
| Middle To Secondary | 0.417 | 0.493 | 0.503 | 0.500 | -0.086** |
| Graduate | 0.106 | 0.308 | 0.112 | 0.316 | -0.006 |
| **Household Size** |  |  |  |  |  |
| > 5 | 0.667 | 0.471 | 0.700 | 0.458 | -0.034* |
| **Place of Birth** |  |  |  |  |  |
| Non-Institutional | 0.308 | 0.462 | 0.146 | 0.353 | 0.163** |
| **Distance to Sub-Center** |  |  |  |  |  |
| < 15 Minutes | 0.255 | 0.436 | 0.243 | 0.429 | 0.012 |
| 15 To 30 Minutes | 0.464 | 0.499 | 0.565 | 0.496 | -0.101** |
| > 30 Minutes | 0.272 | 0.445 | 0.190 | 0.392 | 0.082** |

Note: Data are from INCHIS-1 with 13 districts in intervention group and 8 districts in control group. Numbers are proportion of households within each group. *Full=*1 dose BCG and measles, 3 doses of DPT and polio; *HepB0*=Hepatitis B given at birth, *DPT*=Diphtheria, Pertussis, Tetanus, *OPV=Oral Polio Vaccine,* *OPV0=OPV* birth dose*, BCG*=Bacillus Calmette–Guérin; *Measles1*= First dose of measles; *OTV*= On-time vaccination – considers timely vaccination of DPT and full immunization; Wealth Quintile 1 refers to the poorest wealth quintile; Control group = Children that were not covered by phase 1 or 2 of Mission Indradhanush; +p<0.1, *p<0.05, **p<0.01

**Table S3: Background characteristics of study children by vaccination status (%)**

|  | **Full Immunization** | | **DPT1** | | **DPT2** | | **DPT3** | | **OPV0** | | **OPV1** | | **OPV2** | | **OPV3** | | **Measles** | | **BCG** | | **Hepatitis B** | |
| --- | --- | --- | --- | --- | --- | --- | --- | --- | --- | --- | --- | --- | --- | --- | --- | --- | --- | --- | --- | --- | --- | --- |
|  | **No** | **Yes** | **No** | **Yes** | **No** | **Yes** | **No** | **Yes** | **No** | **Yes** | **No** | **Yes** | **No** | **Yes** | **No** | **Yes** | **No** | **Yes** | **No** | **Yes** | **No** | **Yes** |
| **Sex** |  |  |  |  |  |  |  |  |  |  |  |  |  |  |  |  |  |  |  |  |  |  |
| Male | 28 | 72 | 13 | 87 | 20 | 80 | 32 | 68 | 8 | 92 | 10 | 90 | 21 | 79 | 37 | 63 | 18 | 82 | 5 | 95 | 15 | 85 |
| Female | 36 | 64 | 14 | 86 | 20 | 80 | 35 | 65 | 10 | 90 | 11 | 89 | 23 | 77 | 40 | 60 | 21 | 79 | 6 | 94 | 17 | 83 |
| **Locality** |  |  |  |  |  |  |  |  |  |  |  |  |  |  |  |  |  |  |  |  |  |  |
| Rural | 35 | 65 | 14 | 86 | 20 | 80 | 34 | 66 | 10 | 90 | 11 | 89 | 23 | 77 | 39 | 61 | 21 | 79 | 6 | 94 | 16 | 84 |
| Urban | 25 | 75 | 12 | 88 | 20 | 80 | 33 | 67 | 9 | 91 | 10 | 90 | 20 | 80 | 37 | 63 | 17 | 83 | 6 | 94 | 15 | 85 |
| **Wealth Quintile** |  |  |  |  |  |  |  |  |  |  |  |  |  |  |  |  |  |  |  |  |  |  |
| 1 | 44 | 56 | 27 | 73 | 31 | 69 | 43 | 57 | 16 | 84 | 16 | 84 | 32 | 68 | 47 | 53 | 29 | 71 | 11 | 89 | 25 | 75 |
| 2 | 34 | 66 | 11 | 89 | 19 | 81 | 35 | 65 | 7 | 93 | 10 | 90 | 21 | 79 | 40 | 60 | 22 | 78 | 4 | 96 | 15 | 85 |
| 3 | 29 | 71 | 11 | 89 | 14 | 86 | 25 | 75 | 8 | 92 | 9 | 91 | 19 | 81 | 36 | 64 | 15 | 85 | 4 | 96 | 13 | 87 |
| 4 | 28 | 72 | 10 | 90 | 17 | 83 | 30 | 70 | 8 | 92 | 10 | 90 | 20 | 80 | 35 | 65 | 19 | 81 | 6 | 94 | 11 | 89 |
| 5 | 20 | 80 | 7 | 93 | 18 | 82 | 32 | 68 | 7 | 93 | 9 | 91 | 19 | 81 | 34 | 66 | 10 | 90 | 3 | 97 | 14 | 86 |
| **Religion** |  |  |  |  |  |  |  |  |  |  |  |  |  |  |  |  |  |  |  |  |  |  |
| Hindu | 31 | 69 | 13 | 87 | 19 | 81 | 32 | 68 | 9 | 91 | 10 | 90 | 21 | 79 | 37 | 63 | 20 | 80 | 5 | 95 | 16 | 84 |
| Muslim | 40 | 60 | 20 | 80 | 31 | 69 | 47 | 53 | 14 | 86 | 22 | 78 | 32 | 68 | 48 | 52 | 20 | 80 | 10 | 90 | 19 | 81 |
| Christian | 39 | 61 | 13 | 87 | 19 | 81 | 36 | 64 | 19 | 81 | 5 | 95 | 17 | 83 | 35 | 65 | 18 | 82 | 15 | 85 | 23 | 77 |
| Sikh | 20 | 80 | 7 | 93 | 13 | 87 | 23 | 77 | 8 | 92 | 9 | 91 | 13 | 87 | 30 | 70 | 11 | 89 | 8 | 92 | 7 | 93 |
| Other | 0 | 100 | 0 | 100 | 0 | 100 | 3 | 97 | 10 | 90 | 0 | 100 | 4 | 96 | 4 | 96 | 0 | 100 | 0 | 100 | 17 | 83 |
| **Caste** |  |  |  |  |  |  |  |  |  |  |  |  |  |  |  |  |  |  |  |  |  |  |
| General | 35 | 65 | 15 | 85 | 21 | 79 | 33 | 67 | 11 | 89 | 12 | 88 | 24 | 76 | 43 | 57 | 21 | 79 | 6 | 94 | 21 | 79 |
| Scheduled Tribe | 49 | 51 | 18 | 82 | 24 | 76 | 43 | 57 | 14 | 86 | 8 | 92 | 26 | 74 | 46 | 54 | 19 | 81 | 8 | 92 | 14 | 86 |
| Other Backward Caste | 27 | 73 | 12 | 88 | 18 | 82 | 31 | 69 | 8 | 92 | 11 | 89 | 21 | 79 | 35 | 65 | 16 | 84 | 5 | 95 | 13 | 87 |
| Scheduled Caste | 33 | 67 | 14 | 86 | 21 | 79 | 36 | 64 | 10 | 90 | 10 | 90 | 22 | 78 | 39 | 61 | 24 | 76 | 6 | 94 | 18 | 82 |
| **Education** |  |  |  |  |  |  |  |  |  |  |  |  |  |  |  |  |  |  |  |  |  |  |
| No Schooling | 41 | 59 | 23 | 77 | 29 | 71 | 40 | 60 | 16 | 84 | 17 | 83 | 30 | 70 | 45 | 55 | 22 | 78 | 10 | 90 | 25 | 75 |
| Primary Or Lower | 32 | 68 | 11 | 89 | 17 | 83 | 35 | 65 | 10 | 90 | 10 | 90 | 19 | 81 | 39 | 61 | 25 | 75 | 9 | 91 | 14 | 86 |
| Middle To Secondary | 25 | 75 | 10 | 90 | 16 | 84 | 30 | 70 | 6 | 94 | 8 | 92 | 20 | 80 | 34 | 66 | 17 | 83 | 3 | 97 | 11 | 89 |
| Graduate | 25 | 75 | 3 | 97 | 11 | 89 | 27 | 73 | 5 | 95 | 4 | 96 | 14 | 86 | 34 | 66 | 14 | 86 | 1 | 99 | 11 | 89 |
| **Household size** |  |  |  |  |  |  |  |  |  |  |  |  |  |  |  |  |  |  |  |  |  |  |
| < 5 | 29 | 71 | 11 | 89 | 16 | 84 | 29 | 71 | 6 | 94 | 8 | 92 | 21 | 79 | 37 | 63 | 19 | 81 | 4 | 96 | 12 | 88 |
| > 5 | 32 | 68 | 14 | 86 | 21 | 79 | 35 | 65 | 11 | 89 | 12 | 88 | 22 | 78 | 39 | 61 | 20 | 80 | 6 | 94 | 17 | 83 |
| **Place Of Birth** |  |  |  |  |  |  |  |  |  |  |  |  |  |  |  |  |  |  |  |  |  |  |
| Institutional | 25 | 75 | 10 | 90 | 16 | 84 | 28 | 72 | 6 | 94 | 8 | 92 | 19 | 81 | 34 | 66 | 16 | 84 | 4 | 96 | 12 | 88 |
| Non-Institutional | 60 | 40 | 28 | 72 | 39 | 61 | 57 | 43 | 25 | 75 | 23 | 77 | 38 | 62 | 59 | 41 | 35 | 65 | 16 | 84 | 34 | 66 |
| **Distance to  vaccination site** |  |  |  |  |  |  |  |  |  |  |  |  |  |  |  |  |  |  |  |  |  |  |
| < 15 Minutes | 24 | 76 | 13 | 87 | 19 | 81 | 31 | 69 | 8 | 92 | 11 | 89 | 22 | 78 | 37 | 63 | 18 | 82 | 7 | 93 | 14 | 86 |
| 15 To 30 Minutes | 34 | 66 | 14 | 86 | 21 | 79 | 34 | 66 | 10 | 90 | 10 | 90 | 21 | 79 | 39 | 61 | 20 | 80 | 6 | 94 | 17 | 83 |
| > 30 Minutes | 37 | 63 | 13 | 87 | 20 | 80 | 35 | 65 | 8 | 92 | 12 | 88 | 26 | 74 | 42 | 58 | 22 | 78 | 4 | 96 | 15 | 85 |
|  |  |  |  |  |  |  |  |  |  |  |  |  |  |  |  |  |  |  |  |  |  |  |
| Observations | 7,494 | | 14,892 | | 14,356 | | 13,829 | | 15,304 | | 15,304 | | 15,304 | | 15,304 | | 10,095 | | 15,304 | | 15,304 | |

Note: Data are from INCHIS-1. Numbers are percentages for each binary vaccination outcome e.g., whether fully vaccinated (yes/ no). F*ull=* 1 dose BCG and measles, 3 doses of DPT and polio; *HepB0*=Hepatitis B given at birth, *DPT*=Diphtheria, Pertussis, Tetanus, *OPV=*Oral Polio Vaccine*,* *OPV0=OPV* birth dose*,* *BCG*=Bacillus Calmette–Guérin; *Measles1*= First dose of measles; *OTV*= On-time vaccination – considers timely receipt of all three DPT vaccines and full immunization; Wealth Quintile 1 refers to the poorest wealth quintile.

Table S4: Immunization Outcomes and Phase 1 and 2 Mission Indradhanush Treatment

| Model | 1 | 2 | 3 | 4 | 5 | 6 | 7 | 8 | 9 | 10 | 11 | 12 |
| --- | --- | --- | --- | --- | --- | --- | --- | --- | --- | --- | --- | --- |
| Vaccine | Full | DPT1 | DPT2 | DPT3 | OPV0 | OPV1 | OPV2 | OPV3 | DPT3 | BCG | HepB0 | OTV |
|  |  |  |  |  |  |  |  |  |  |  |  |  |
| *Phase 1&2=1* | -0.23** | -0.04 | -0.06* | -0.11+ | -0.01 | -0.07* | -0.08** | -0.08* | -0.01 | -0.05* | -0.07+ | -0.07* |
|  | 0.03 | 0.03 | 0.02 | 0.06 | 0.02 | 0.03 | 0.03 | 0.03 | 0.05 | 0.02 | 0.04 | 0.03 |
|  |  |  |  |  |  |  |  |  |  |  |  |  |
| *Post-Intervention=1* | -0.02 | 0.03 | 0.02 | 0.03 | 0 | 0 | 0.03 | 0.04 | 0.05 | 0 | -0.11** | -0.02 |
|  | 0.03 | 0.02 | 0.03 | 0.05 | 0.02 | 0.01 | 0.02 | 0.04 | 0.03 | 0.01 | 0.03 | 0.04 |
|  |  |  |  |  |  |  |  |  |  |  |  |  |
| *DID Indicator* | 0.27** | 0.02 | 0.07+ | 0.15+ | 0.09* | 0.09** | 0.11* | 0.16** | 0.05 | 0.05* | 0.19** | 0.08* |
|  | 0.08 | 0.03 | 0.04 | 0.08 | 0.03 | 0.02 | 0.04 | 0.06 | 0.05 | 0.02 | 0.04 | 0.04 |
|  |  |  |  |  |  |  |  |  |  |  |  |  |
| Age of Mother | 0 | 0 | 0 | 0 | 0 | 0 | 0 | 0 | 0 | 0 | 0 | -0.00+ |
|  | 0 | 0 | 0 | 0 | 0 | 0 | 0 | 0 | 0 | 0 | 0 | 0 |
|  |  |  |  |  |  |  |  |  |  |  |  |  |
| *Sex (Male=1)* | | | | | | | | | | | | |
| Female | -0.06+ | 0 | 0.01 | 0 | -0.01 | 0 | 0 | -0.02 | -0.02 | 0 | -0.01 | 0.01 |
|  | 0.03 | 0.01 | 0.02 | 0.02 | 0.01 | 0.01 | 0.01 | 0.02 | 0.02 | 0.01 | 0.02 | 0.02 |
|  |  |  |  |  |  |  |  |  |  |  |  |  |
| *Locality (Rural=1)* | | | | | | | | | | | | |
| Urban | 0.01 | -0.01 | -0.02 | 0 | 0.01 | 0.01 | 0 | 0.01 | 0 | -0.01 | 0.02 | 0.01 |
|  | 0.03 | 0.02 | 0.02 | 0.02 | 0.01 | 0.01 | 0.01 | 0.02 | 0.02 | 0.01 | 0.01 | 0.02 |
|  |  |  |  |  |  |  |  |  |  |  |  |  |
| Age of Child | 0 | 0.00** | 0.00** | 0.00** | 0.00* | 0.00** | 0.00** | 0.01** | 0.00** | 0.00** | 0.00** | 0.01** |
|  | 0 | 0 | 0 | 0 | 0 | 0 | 0 | 0 | 0 | 0 | 0 | 0 |
|  |  |  |  |  |  |  |  |  |  |  |  |  |
| *Wealth Quintile (1=1)* | | | | | | | | | | | | |
| 2 | 0.06** | 0.08** | 0.06 | 0.05** | 0.05** | 0.05** | 0.08* | 0.06** | 0.04+ | 0.03+ | 0.05+ | 0.02 |
|  | 0.02 | 0.03 | 0.04 | 0.02 | 0.02 | 0.02 | 0.03 | 0.02 | 0.02 | 0.02 | 0.03 | 0.02 |
| 3 | 0.09** | 0.06+ | 0.06 | 0.06** | 0.03 | 0.03 | 0.07* | 0.06* | 0.05** | 0.02 | 0.03 | 0.02 |
|  | 0.02 | 0.03 | 0.04 | 0.02 | 0.02 | 0.02 | 0.03 | 0.03 | 0.02 | 0.02 | 0.03 | 0.03 |
| 4 | 0.11** | 0.07* | 0.05 | 0.05+ | 0.02 | 0.03 | 0.06+ | 0.09** | 0.06+ | 0.01 | 0.05 | 0.05 |
|  | 0.02 | 0.03 | 0.04 | 0.03 | 0.02 | 0.02 | 0.04 | 0.03 | 0.03 | 0.03 | 0.03 | 0.03 |
| 5 | 0.17** | 0.09* | 0.06 | 0.07* | 0.05 | 0.06+ | 0.09* | 0.12** | 0.11* | 0.05+ | 0.05 | 0.04+ |
|  | 0.03 | 0.04 | 0.04 | 0.03 | 0.03 | 0.03 | 0.03 | 0.02 | 0.04 | 0.03 | 0.03 | 0.02 |
|  |  |  |  |  |  |  |  |  |  |  |  |  |
| *Religion (Hindu=1)* | | | | | | | | | | | | |
| Muslim | 0.01 | 0 | -0.02 | 0 | 0 | -0.02 | -0.01 | 0 | -0.02 | 0 | 0.04+ | -0.01 |
|  | 0.02 | 0.03 | 0.02 | 0.03 | 0.02 | 0.03 | 0.02 | 0.02 | 0.03 | 0.02 | 0.02 | 0.03 |
| Christian | 0.1 | 0.01 | 0.02 | 0.05 | 0.02 | 0.01 | 0 | 0.04 | 0.05 | -0.03 | 0.03 | -0.06 |
|  | 0.06 | 0.02 | 0.03 | 0.04 | 0.02 | 0.03 | 0.03 | 0.04 | 0.04 | 0.03 | 0.04 | 0.06 |
| Sikh | -0.03 | 0 | -0.03 | 0 | -0.01 | -0.04 | -0.01 | -0.05 | 0.03 | -0.03 | 0.01 | 0.07 |
|  | 0.04 | 0.02 | 0.05 | 0.04 | 0.03 | 0.03 | 0.03 | 0.05 | 0.03 | 0.03 | 0.02 | 0.08 |
| Other | 0.01 | 0 | 0.01 | 0.02 | 0.02 | -0.01 | -0.02 | 0.01 | -0.06 | -0.01 | -0.04 | -0.10* |
|  | 0.05 | 0.01 | 0.01 | 0.04 | 0.02 | 0.02 | 0.03 | 0.05 | 0.04 | 0.01 | 0.06 | 0.04 |
|  |  |  |  |  |  |  |  |  |  |  |  |  |
| *Caste (General=1)* | | | | | | | | | | | | |
| Scheduled tribe | -0.02 | 0.02 | 0.01 | -0.03 | -0.01 | 0.02 | 0 | -0.01 | 0.01 | 0 | 0.01 | 0 |
|  | 0.05 | 0.02 | 0.02 | 0.04 | 0.02 | 0.01 | 0.03 | 0.03 | 0.03 | 0.01 | 0.02 | 0.03 |
| Other backward caste | 0.07+ | 0.03+ | 0.02 | 0.01 | 0.02+ | 0.02 | 0.03 | 0.06* | 0.03 | 0 | 0.04* | 0.04 |
|  | 0.04 | 0.01 | 0.02 | 0.03 | 0.01 | 0.01 | 0.02 | 0.03 | 0.03 | 0.01 | 0.02 | 0.02 |
| Scheduled caste | 0.09** | 0.07** | 0.05* | 0.04 | 0.04+ | 0.05* | 0.05+ | 0.07** | 0.05* | 0.02+ | 0.05+ | 0.01 |
|  | 0.03 | 0.02 | 0.02 | 0.03 | 0.02 | 0.02 | 0.03 | 0.03 | 0.02 | 0.01 | 0.02 | 0.02 |
|  |  |  |  |  |  |  |  |  |  |  |  |  |
| *No schooling* | | | | | | | | | | | | |
| Primary or lower | 0.06* | 0.04** | 0.04** | 0.01 | 0.02 | 0.03** | 0.04* | 0.03 | -0.03 | 0 | 0.05* | 0.07** |
|  | 0.03 | 0.01 | 0.01 | 0.02 | 0.02 | 0.01 | 0.02 | 0.02 | 0.03 | 0.01 | 0.02 | 0.02 |
| Middle to Secondary | 0.07** | 0.05** | 0.05* | 0.03 | 0.04* | 0.04* | 0.04* | 0.04* | 0 | 0.03* | 0.05* | 0.09** |
|  | 0.03 | 0.02 | 0.02 | 0.02 | 0.02 | 0.02 | 0.02 | 0.02 | 0.02 | 0.01 | 0.02 | 0.02 |
| Graduate | 0.06+ | 0.09** | 0.09* | 0.04 | 0.05 | 0.07* | 0.08** | 0.05* | 0 | 0.04+ | 0.08* | 0.13** |
|  | 0.03 | 0.03 | 0.03 | 0.03 | 0.03 | 0.03 | 0.03 | 0.02 | 0.03 | 0.02 | 0.03 | 0.04 |
|  |  |  |  |  |  |  |  |  |  |  |  |  |
| *Household size (<5=1)* | |  |  |  |  |  |  |  |  |  |  |  |
| > 5 | 0 | -0.01 | -0.01 | 0 | 0 | 0.01 | 0.02 | 0.03 | 0.01 | 0 | 0.01 | 0.02 |
|  | 0.03 | 0.01 | 0.01 | 0.02 | 0.01 | 0.01 | 0.01 | 0.02 | 0.02 | 0.01 | 0.01 | 0.02 |
|  |  |  |  |  |  |  |  |  |  |  |  |  |
| *Institutional Delivery (Institutional=1)* | | | | | | | | | | | | |
| Non-Institutional | -0.17** | -0.09** | -0.13** | -0.13** | -0.14** | -0.07** | -0.09** | -0.12** | -0.13** | -0.07** | -0.15** | -0.08** |
|  | 0.04 | 0.02 | 0.03 | 0.02 | 0.02 | 0.01 | 0.02 | 0.02 | 0.04 | 0.01 | 0.03 | 0.02 |
|  |  |  |  |  |  |  |  |  |  |  |  |  |
| *Distance to Vaccination Site (< 15 minutes=1)* | | | | | | | | | | | | |
| 15 to 30 minutes | -0.05+ | -0.02+ | -0.02 | -0.03 | 0 | 0 | -0.01 | -0.01 | 0 | 0 | 0 | -0.04* |
|  | 0.03 | 0.01 | 0.01 | 0.02 | 0.01 | 0.01 | 0.02 | 0.01 | 0.02 | 0.01 | 0.01 | 0.02 |
| > 30 minutes | -0.11* | -0.03** | -0.05* | -0.06* | 0.01 | -0.02 | -0.05** | -0.07* | -0.05 | 0.01 | 0 | -0.05+ |
|  | 0.04 | 0.01 | 0.02 | 0.03 | 0.02 | 0.01 | 0.02 | 0.03 | 0.04 | 0.01 | 0.02 | 0.03 |
|  |  |  |  |  |  |  |  |  |  |  |  |  |
| Observations | 4474 | 8603 | 8272 | 7917 | 9033 | 8699 | 8501 | 8282 | 5651 | 9033 | 9033 | 8315 |
| Pseudo R^2^ | 0.155 | 0.107 | 0.133 | 0.164 | 0.131 | 0.126 | 0.183 | 0.219 | 0.129 | 0.1 | 0.167 | 0.178 |

Note: *Full=* 1 dose BCG and measles, 3 doses of DPT and polio*; HepB0*=Hepatitis B given at birth, *DPT*=Diphtheria, Pertussis, Tetanus, *BCG*=Bacillus Calmette–Guérin; *OPV=Oral Polio Vaccine; OPV0=OPV* birth dose*;*  *Measles1*= First dose of measles; *OTV*= On-time vaccination – considers timely vaccination of DPT and full immunization.*; Phase 1 and 2 control*: did not receive treatment in phase 1 or phase 2; +p-value<0.10, *p-value<0.05, **p-value<0.01; Standard errors below coefficients.

Table S5: Immunization Outcomes and Phase 1 Mission Indradhanush Treatment

| Model | 1 | 2 | 3 | 4 | 5 | 6 | 7 | 8 | 9 | 10 | 11 | 12 |
| --- | --- | --- | --- | --- | --- | --- | --- | --- | --- | --- | --- | --- |
| Vaccine | Full | DPT1 | DPT2 | DPT3 | OPV0 | OPV1 | OPV2 | OPV3 | DPT3 | BCG | HepB0 | OTV |
|  |  |  |  |  |  |  |  |  |  |  |  |  |
| *Phase 1&2=1* | -0.11** | -0.04+ | -0.04* | -0.08* | -0.04* | -0.03* | -0.03+ | -0.06* | -0.02 | -0.03+ | -0.05* | -0.07** |
|  | 0.04 | 0.02 | 0.02 | 0.03 | 0.02 | 0.02 | 0.02 | 0.03 | 0.03 | 0.02 | 0.02 | 0.03 |
|  |  |  |  |  |  |  |  |  |  |  |  |  |
| *Post-Intervention=1* | 0.09* | 0.06** | 0.08** | 0.10** | 0.02* | 0.04** | 0.11** | 0.14** | 0.09** | 0.03** | 0.01 | 0.02 |
|  | 0.04 | 0.01 | 0.01 | 0.03 | 0.01 | 0.01 | 0.01 | 0.02 | 0.03 | 0.01 | 0.02 | 0.03 |
|  |  |  |  |  |  |  |  |  |  |  |  |  |
| *DID Indicator* | 0.04 | 0 | 0 | 0.02 | 0.01 | 0 | -0.01 | 0 | -0.04 | 0.01 | 0.01 | 0.01 |
|  | 0.05 | 0.03 | 0.03 | 0.04 | 0.02 | 0.02 | 0.02 | 0.04 | 0.03 | 0.02 | 0.04 | 0.03 |
|  |  |  |  |  |  |  |  |  |  |  |  |  |
| Age of Mother | 0 | 0 | 0 | 0 | 0 | 0 | 0 | 0 | 0 | 0 | 0 | 0 |
|  | 0 | 0 | 0 | 0 | 0 | 0 | 0 | 0 | 0 | 0 | 0 | 0 |
|  |  |  |  |  |  |  |  |  |  |  |  |  |
| *Sex (Male=1)* | | | | | | | | | | | | |
| Female | 0.01 | 0 | 0.01 | 0 | 0 | 0 | 0.01 | -0.01 | 0.01 | 0 | 0 | -0.01 |
|  | 0.02 | 0.01 | 0.01 | 0.01 | 0.01 | 0.01 | 0.01 | 0.01 | 0.01 | 0 | 0.01 | 0.01 |
|  |  |  |  |  |  |  |  |  |  |  |  |  |
| *Locality (Rural=1)* | | | | | | | | | | | | |
| Urban | 0.03 | -0.01 | -0.01 | 0.01 | -0.01 | -0.01 | -0.01 | 0.02 | 0 | -0.01+ | -0.02 | 0.02+ |
|  | 0.02 | 0.01 | 0.01 | 0.02 | 0.01 | 0.01 | 0.01 | 0.01 | 0.01 | 0.01 | 0.01 | 0.01 |
|  |  |  |  |  |  |  |  |  |  |  |  |  |
| Age of Child | 0 | 0.00** | 0.00** | 0.00** | 0.00** | 0.00** | 0.00** | 0.01** | 0.00** | 0.00** | 0.00** | 0.00** |
|  | 0 | 0 | 0 | 0 | 0 | 0 | 0 | 0 | 0 | 0 | 0 | 0 |
|  |  |  |  |  |  |  |  |  |  |  |  |  |
| *Wealth Quintile (1=1)* | | | | | | | | | | | | |
| 2 | 0.06** | 0.05** | 0.04** | 0.04* | 0 | 0.04** | 0.04** | 0.04** | 0.03+ | 0.02 | 0.05** | 0.03* |
|  | 0.02 | 0.01 | 0.01 | 0.02 | 0.01 | 0.01 | 0.01 | 0.01 | 0.01 | 0.01 | 0.02 | 0.02 |
| 3 | 0.03 | 0.04** | 0.04* | 0.02 | 0 | 0.05** | 0.05** | 0.03+ | 0.01 | 0.02+ | 0.03 | 0.03* |
|  | 0.03 | 0.02 | 0.02 | 0.02 | 0.02 | 0.01 | 0.01 | 0.02 | 0.02 | 0.01 | 0.02 | 0.01 |
| 4 | 0.06* | 0.06** | 0.07** | 0.05* | 0.01 | 0.05** | 0.06** | 0.04+ | 0.02 | 0.04** | 0.07** | 0.04* |
|  | 0.03 | 0.02 | 0.02 | 0.02 | 0.02 | 0.02 | 0.02 | 0.02 | 0.02 | 0.01 | 0.02 | 0.02 |
| 5 | 0.11** | 0.08** | 0.08** | 0.08** | 0.01 | 0.07** | 0.07** | 0.08** | 0.07** | 0.05** | 0.08** | 0.07** |
|  | 0.03 | 0.02 | 0.03 | 0.02 | 0.03 | 0.02 | 0.02 | 0.02 | 0.02 | 0.01 | 0.03 | 0.02 |
|  |  |  |  |  |  |  |  |  |  |  |  |  |
| *Religion (Hindu=1)* | | | | | | | | | | | | |
| Muslim | -0.08** | -0.03 | -0.06* | -0.06* | -0.03 | -0.02 | -0.05* | -0.06* | -0.08** | -0.03 | -0.05** | -0.05** |
|  | 0.03 | 0.02 | 0.02 | 0.03 | 0.03 | 0.02 | 0.02 | 0.02 | 0.03 | 0.02 | 0.02 | 0.01 |
| Christian | -0.06 | 0 | -0.02 | 0.01 | 0.01 | 0.02+ | 0.01 | 0 | -0.04 | 0 | -0.01 | 0 |
|  | 0.05 | 0.01 | 0.02 | 0.03 | 0.01 | 0.01 | 0.01 | 0.03 | 0.03 | 0.01 | 0.03 | 0.03 |
| Sikh | 0.09* | 0.03+ | 0.01 | 0.03 | 0.03* | -0.02 | 0.02 | 0 | 0.03 | 0 | 0.02 | 0.08 |
|  | 0.04 | 0.02 | 0.04 | 0.03 | 0.01 | 0.02 | 0.03 | 0.04 | 0.02 | 0.01 | 0.02 | 0.08 |
| Other | -0.02 | 0.01 | -0.02 | -0.01 | 0 | -0.01 | -0.04* | -0.01 | -0.06 | -0.02+ | -0.05 | -0.07 |
|  | 0.05 | 0.01 | 0.02 | 0.03 | 0.01 | 0.01 | 0.02 | 0.02 | 0.04 | 0.01 | 0.03 | 0.05 |
|  |  |  |  |  |  |  |  |  |  |  |  |  |
| *Caste (General=1)* | | | | | | | | | | | | |
| Scheduled tribe | -0.04 | 0.02 | 0.02 | -0.06+ | 0.01 | 0.03 | -0.01 | -0.06+ | 0.02 | 0.03+ | 0.04* | -0.06* |
|  | 0.05 | 0.02 | 0.03 | 0.03 | 0.02 | 0.02 | 0.02 | 0.03 | 0.03 | 0.02 | 0.02 | 0.03 |
| Other backward caste | 0.02 | 0.02 | 0.01 | 0 | 0 | 0.02 | 0.01 | 0.01 | 0.03 | 0.02 | 0.03 | -0.01 |
|  | 0.03 | 0.02 | 0.02 | 0.02 | 0.02 | 0.01 | 0.02 | 0.02 | 0.02 | 0.02 | 0.02 | 0.02 |
| Scheduled caste | 0.01 | 0.05** | 0.02 | 0 | 0.01 | 0.03+ | 0.02 | 0 | 0.03 | 0.02+ | 0.05** | -0.02 |
|  | 0.02 | 0.02 | 0.02 | 0.02 | 0.02 | 0.01 | 0.02 | 0.02 | 0.02 | 0.01 | 0.02 | 0.02 |
|  |  |  |  |  |  |  |  |  |  |  |  |  |
| *No schooling* | | | | | | | | | | | | |
| Primary or lower | 0.04+ | 0.02* | 0.05** | 0.04** | 0.02 | 0.02* | 0.04** | 0.05** | 0.04* | 0.02* | 0.05** | 0.04** |
|  | 0.02 | 0.01 | 0.01 | 0.01 | 0.01 | 0.01 | 0.01 | 0.01 | 0.02 | 0.01 | 0.02 | 0.01 |
| Middle to Secondary | 0.03+ | 0.04** | 0.05** | 0.04** | 0.02 | 0.02 | 0.04* | 0.04** | 0.04** | 0.02* | 0.03* | 0.04** |
|  | 0.02 | 0.01 | 0.02 | 0.01 | 0.01 | 0.01 | 0.01 | 0.02 | 0.01 | 0.01 | 0.02 | 0.02 |
| Graduate | 0.04 | 0.07** | 0.08** | 0.06** | 0.05** | 0.04* | 0.06** | 0.04* | 0.08** | 0.04** | 0.07* | 0.07** |
|  | 0.03 | 0.02 | 0.02 | 0.02 | 0.02 | 0.02 | 0.02 | 0.02 | 0.02 | 0.01 | 0.03 | 0.02 |
|  |  |  |  |  |  |  |  |  |  |  |  |  |
| *Household size (<5=1)* | |  |  |  |  |  |  |  |  |  |  |  |
| > 5 | 0 | -0.01 | 0 | 0.01 | -0.02* | -0.01 | 0.01 | 0.01 | 0 | -0.01 | -0.01 | -0.02 |
|  | 0.02 | 0.01 | 0.01 | 0.01 | 0.01 | 0.01 | 0.01 | 0.01 | 0.01 | 0 | 0.01 | 0.01 |
|  |  |  |  |  |  |  |  |  |  |  |  |  |
| *Institutional Delivery (Institutional=1)* | | | | | | | | | | | | |
| Non-Institutional | -0.11** | -0.06** | -0.08** | -0.10** | -0.11** | -0.08** | -0.08** | -0.11** | -0.09** | -0.07** | -0.13** | -0.04** |
|  | 0.03 | 0.02 | 0.02 | 0.02 | 0.02 | 0.01 | 0.02 | 0.02 | 0.02 | 0.02 | 0.02 | 0.01 |
|  |  |  |  |  |  |  |  |  |  |  |  |  |
| *Distance to Vaccination Site (< 15 minutes=1)* | | | | | | | | | | | | |
| 15 to 30 minutes | -0.06** | -0.02 | -0.02 | -0.02+ | 0 | 0 | -0.01 | -0.03* | -0.04* | 0 | 0 | -0.02* |
|  | 0.02 | 0.01 | 0.01 | 0.01 | 0.01 | 0.01 | 0.01 | 0.01 | 0.02 | 0.01 | 0.01 | 0.01 |
| > 30 minutes | -0.10** | -0.07** | -0.06** | -0.07** | -0.02 | -0.05** | -0.07** | -0.08** | -0.07** | -0.02 | -0.02 | -0.03 |
|  | 0.03 | 0.01 | 0.02 | 0.02 | 0.02 | 0.02 | 0.02 | 0.02 | 0.02 | 0.01 | 0.02 | 0.02 |
|  |  |  |  |  |  |  |  |  |  |  |  |  |
| Observations | 8813 | 17720 | 17036 | 16388 | 18574 | 17931 | 17530 | 17166 | 11793 | 18574 | 18574 | 17068 |
| Pseudo R^2^ | 0.133 | 0.105 | 0.138 | 0.151 | 0.121 | 0.13 | 0.208 | 0.234 | 0.141 | 0.098 | 0.173 | 0.147 |

Note: Analysis based on INCHIS-1 and INCHIS-2. *Full=* 1 dose BCG and measles, 3 doses of DPT and polio*; HepB0*=Hepatitis B given at birth, *DPT*=Diphtheria, Pertussis, Tetanus, *BCG*=Bacillus Calmette–Guérin; *OPV=Oral Polio Vaccine; OPV0=OPV* birth dose*;*  *Measles1*= First dose of measles; *OTV*= On-time vaccination – considers timely vaccination of DPT and full immunization.; +p-value<0.10, *p-value<0.05, **p-value<0.01; Standard errors below coefficients.

Table S6: Immunization Outcomes and Phase 2 Mission Indradhanush Treatment

| Model | 1 | 2 | 3 | 4 | 5 | 6 | 7 | 8 | 9 | 10 | 11 | 12 |
| --- | --- | --- | --- | --- | --- | --- | --- | --- | --- | --- | --- | --- |
| Vaccine | Full | DPT1 | DPT2 | DPT3 | OPV0 | OPV1 | OPV2 | OPV3 | DPT3 | BCG | HepB0 | OTV |
|  |  |  |  |  |  |  |  |  |  |  |  |  |
| *Phase 1&2=1* | -0.04 | -0.02 | -0.03+ | -0.05** | -0.02 | -0.02+ | -0.03 | -0.04** | -0.02 | 0 | -0.03+ | -0.04+ |
|  | 0.02 | 0.01 | 0.02 | 0.02 | 0.01 | 0.01 | 0.02 | 0.01 | 0.01 | 0.01 | 0.02 | 0.02 |
|  |  |  |  |  |  |  |  |  |  |  |  |  |
| *Post-Intervention=1* | -0.11** | -0.02 | -0.05* | -0.08** | -0.02 | -0.02 | -0.04+ | -0.08** | -0.03 | -0.01 | -0.07** | 0 |
|  | 0.03 | 0.01 | 0.03 | 0.03 | 0.01 | 0.01 | 0.03 | 0.02 | 0.03 | 0.01 | 0.02 | 0.03 |
|  |  |  |  |  |  |  |  |  |  |  |  |  |
| *DID Indicator* | 0 | 0.01 | 0.02 | 0.01 | 0.03 | 0.01 | 0.02 | 0.01 | 0 | 0 | 0.04 | -0.04 |
|  | 0.04 | 0.02 | 0.03 | 0.03 | 0.02 | 0.02 | 0.03 | 0.03 | 0.02 | 0.01 | 0.03 | 0.03 |
|  |  |  |  |  |  |  |  |  |  |  |  |  |
| Age of Mother | 0.00+ | 0 | 0 | 0.00** | -0.00+ | 0 | 0 | 0.00+ | 0 | -0.00+ | 0 | 0 |
|  | 0 | 0 | 0 | 0 | 0 | 0 | 0 | 0 | 0 | 0 | ^6^0 | 0 |
|  |  |  |  |  |  |  |  |  |  |  |  |  |
| *Sex (Male=1)* | | | | | | | | | | | | |
| Female | 0.02+ | 0 | 0 | 0 | 0.01 | 0 | -0.01+ | -0.01 | 0 | 0 | 0 | 0.01 |
|  | 0.01 | 0 | 0.01 | 0.01 | 0 | 0 | 0.01 | 0.01 | 0.01 | 0 | 0.01 | 0.01 |
|  |  |  |  |  |  |  |  |  |  |  |  |  |
| *Locality (Rural=1)* | | | | | | | | | | | | |
| Urban | -0.01 | -0.02* | -0.03** | -0.02 | 0 | -0.01 | -0.02+ | 0 | -0.04** | -0.01 | -0.01 | 0.01 |
|  | 0.02 | 0.01 | 0.01 | 0.02 | 0.01 | 0.01 | 0.01 | 0.02 | 0.01 | 0.01 | 0.01 | 0.02 |
|  |  |  |  |  |  |  |  |  |  |  |  |  |
| Age of Child | 0 | 0 | 0.00** | 0.00** | 0 | 0 | 0.00** | 0.00** | 0.00** | 0 | 0.00* | 0.01** |
|  | 0 | 0 | 0 | 0 | 0 | 0 | 0 | 0 | 0 | 0 | 0 | 0 |
|  |  |  |  |  |  |  |  |  |  |  |  |  |
| *Wealth Quintile (1=1)* | | | | | | | | | | | | |
| 2 | 0.02 | 0.02+ | 0.02 | 0.02 | 0.04** | 0.02* | 0.02 | 0.01 | 0.03** | 0.01 | 0.02 | 0.01 |
|  | 0.02 | 0.01 | 0.01 | 0.02 | 0.01 | 0.01 | 0.01 | 0.02 | 0.01 | 0.01 | 0.01 | 0.02 |
| 3 | 0.05+ | 0.02 | 0.01 | 0.02 | 0.04** | 0.02* | 0.01 | 0.04* | 0.02 | 0.02+ | 0.01 | 0.02 |
|  | 0.03 | 0.01 | 0.02 | 0.02 | 0.01 | 0.01 | 0.01 | 0.02 | 0.02 | 0.01 | 0.02 | 0.02 |
| 4 | 0.04 | 0.02 | 0.04+ | 0.04 | 0.05** | 0.02* | 0.03* | 0.05* | 0.03+ | 0.02* | 0.02 | 0.03 |
|  | 0.03 | 0.01 | 0.02 | 0.02 | 0.01 | 0.01 | 0.01 | 0.02 | 0.02 | 0.01 | 0.02 | 0.02 |
| 5 | 0.08** | 0.03+ | 0.05* | 0.06* | 0.05** | 0.02+ | 0.04** | 0.07** | 0.05** | 0.02* | 0.03 | 0.08** |
|  | 0.03 | 0.02 | 0.02 | 0.02 | 0.01 | 0.01 | 0.01 | 0.02 | 0.02 | 0.01 | 0.02 | 0.02 |
|  |  |  |  |  |  |  |  |  |  |  |  |  |
| *Religion (Hindu=1)* | | | | | | | | | | | | |
| Muslim | -0.02 | -0.01 | -0.02 | -0.02 | 0.01 | 0 | 0 | -0.03 | 0 | 0 | 0.01 | 0 |
|  | 0.03 | 0.01 | 0.02 | 0.02 | 0.01 | 0.01 | 0.01 | 0.02 | 0.01 | 0.01 | 0.01 | 0.02 |
| Christian | -0.06 | 0.01 | 0.01 | 0 | 0 | 0 | 0 | 0 | -0.03 | 0 | 0 | -0.05 |
|  | 0.05 | 0.01 | 0.01 | 0.02 | 0.01 | 0.02 | 0.02 | 0.02 | 0.03 | 0.01 | 0.02 | 0.04 |
| Sikh | 0.07** | 0 | -0.01 | 0.02 | 0.01 | 0 | -0.01 | 0.01 | 0.02+ | 0.01 | 0.02** | 0.08* |
|  | 0.02 | 0.01 | 0.02 | 0.02 | 0.01 | 0.01 | 0.01 | 0.02 | 0.01 | 0.01 | 0.01 | 0.04 |
| Other | -0.04 | 0.01* | 0.02+ | 0.01 | 0.02 | 0 | 0.01 | 0.01 | -0.08* | 0 | -0.05 | -0.08* |
|  | 0.04 | 0.01 | 0.01 | 0.03 | 0.01 | 0.01 | 0.02 | 0.02 | 0.04 | 0 | 0.04 | 0.03 |
|  |  |  |  |  |  |  |  |  |  |  |  |  |
| *Caste (General=1)* | | | | | | | | | | | | |
| Scheduled tribe | -0.08* | -0.02 | -0.01 | -0.04+ | -0.03* | -0.03** | -0.02+ | -0.06** | -0.04** | -0.01+ | -0.05+ | -0.03 |
|  | 0.03 | 0.01 | 0.02 | 0.02 | 0.01 | 0.01 | 0.01 | 0.02 | 0.01 | 0.01 | 0.02 | 0.02 |
| Other backward caste | -0.02 | 0 | 0 | -0.01 | 0 | 0 | 0 | 0 | -0.02 | 0 | 0 | 0 |
|  | 0.03 | 0.01 | 0.01 | 0.01 | 0.01 | 0 | 0.01 | 0.01 | 0.01 | 0 | 0.01 | 0.02 |
| Scheduled caste | -0.01 | 0.01 | 0 | -0.03+ | -0.01 | 0 | 0 | -0.02 | 0 | 0 | 0 | -0.02 |
|  | 0.02 | 0.01 | 0.01 | 0.02 | 0.01 | 0.01 | 0.01 | 0.02 | 0.01 | 0 | 0.01 | 0.02 |
|  |  |  |  |  |  |  |  |  |  |  |  |  |
| *No schooling* | | | | | | | | | | | | |
| Primary or lower | 0.06** | 0.02+ | 0.03+ | 0.03+ | 0 | 0.01 | 0.02 | 0.03+ | 0.04** | 0.01** | 0.02 | 0.02 |
|  | 0.02 | 0.01 | 0.01 | 0.02 | 0 | 0.01 | 0.01 | 0.02 | 0.01 | 0 | 0.01 | 0.01 |
| Middle to Secondary | 0.07** | 0.04** | 0.05** | 0.07** | 0 | 0.02* | 0.03* | 0.05** | 0.04** | 0.02** | -0.01 | 0.07** |
|  | 0.02 | 0.01 | 0.01 | 0.02 | 0.01 | 0.01 | 0.01 | 0.02 | 0.01 | 0 | 0.01 | 0.02 |
| Graduate | 0.10** | 0.05** | 0.06** | 0.08** | 0.01 | 0.02* | 0.03+ | 0.02 | 0.06** | 0.02** | 0.01 | 0.05+ |
|  | 0.03 | 0.01 | 0.02 | 0.03 | 0.01 | 0.01 | 0.02 | 0.02 | 0.01 | 0.01 | 0.01 | 0.03 |
|  |  |  |  |  |  |  |  |  |  |  |  |  |
| *Household size (<5=1)* | |  |  |  |  |  |  |  |  |  |  |  |
| > 5 | -0.01 | 0 | 0 | 0 | 0 | 0 | -0.01+ | 0 | -0.01 | -0.01* | 0 | 0 |
|  | 0.02 | 0.01 | 0.01 | 0.01 | 0.01 | 0 | 0 | 0.01 | 0.01 | 0 | 0.01 | 0.01 |
|  |  |  |  |  |  |  |  |  |  |  |  |  |
| *Institutional Delivery (Institutional=1)* | | | | | | | | | | | | |
| Non-Institutional | -0.11** | -0.03+ | -0.04+ | -0.04 | -0.08** | -0.04* | -0.05** | -0.06* | -0.09** | -0.05** | -0.16** | -0.05** |
|  | 0.03 | 0.02 | 0.02 | 0.02 | 0.02 | 0.02 | 0.02 | 0.02 | 0.03 | 0.01 | 0.03 | 0.01 |
|  |  |  |  |  |  |  |  |  |  |  |  |  |
| *Distance to Vaccination Site (< 15 minutes=1)* | | | | | | | | | | | | |
| 15 to 30 minutes | -0.03+ | -0.01 | -0.01 | -0.01 | 0.01 | 0 | 0 | -0.01 | 0 | 0 | 0.01 | -0.04** |
|  | 0.02 | 0.01 | 0.01 | 0.01 | 0.01 | 0.01 | 0.01 | 0.01 | 0.01 | 0 | 0.01 | 0.02 |
| > 30 minutes | -0.05* | -0.03+ | -0.04* | -0.02 | 0 | -0.02 | -0.03 | -0.04* | -0.01 | 0 | -0.01 | -0.04 |
|  | 0.02 | 0.02 | 0.02 | 0.02 | 0.01 | 0.01 | 0.02 | 0.02 | 0.02 | 0.01 | 0.02 | 0.02 |
|  |  |  |  |  |  |  |  |  |  |  |  |  |
| Observations | 6770 | 12973 | 12454 | 11973 | 13786 | 12973 | 12454 | 11973 | 8602 | 13786 | 13786 | 12595 |
| Pseudo R^2^ | 0.077 | 0.041 | 0.05 | 0.072 | 0.071 | 0.047 | 0.065 | 0.093 | 0.061 | 0.045 | 0.111 | 0.19 |

Note: *Full=* 1 dose BCG and measles, 3 doses of DPT and polio*; HepB0*=Hepatitis B given at birth, *DPT*=Diphtheria, Pertussis, Tetanus, *BCG*=Bacillus Calmette–Guérin; *OPV=Oral Polio Vaccine; OPV0=OPV* birth dose*;*  *Measles1*: First dose of measles; *OTV*: On-time vaccination – considers timely vaccination of DPT and full immunization.; +p-value<0.10, *p-value<0.05, **p-value<0.01; Standard errors below coefficients.

Table S7: Immunization Outcomes and Phase 1 and 2 Mission Indradhanush Treatment, Vaccination Card Seen

| Model | 1 | 2 | 3 | 4 | 5 | 6 | 7 | 8 | 9 | 10 | 11 | 12 |
| --- | --- | --- | --- | --- | --- | --- | --- | --- | --- | --- | --- | --- |
| Vaccine | Full | DPT1 | DPT2 | DPT3 | OPV0 | OPV1 | OPV2 | OPV3 | DPT3 | BCG | HepB0 | OTV |
|  |  |  |  |  |  |  |  |  |  |  |  |  |
| *Phase 1&2=1* | -0.73** | -0.06 | -0.16 | -0.36+ | 0.23+ | -0.24 | -0.25* | -0.23* | 0.02 | -0.66* | -0.29* | -0.14 |
|  | 0.19 | 0.13 | 0.1 | 0.18 | 0.13 | 0.18 | 0.13 | 0.1 | 0.16 | 0.26 | 0.13 | 0.15 |
|  |  |  |  |  |  |  |  |  |  |  |  |  |
| *Post-Intervention=1* | -0.08 | 0.27 | 0.06 | 0.1 | -0.09 | -0.05 | -0.04 | 0.04 | 0.25+ | -0.35+ | -0.66** | -0.07 |
|  | 0.15 | 0.18 | 0.15 | 0.18 | 0.22 | 0.18 | 0.13 | 0.15 | 0.13 | 0.19 | 0.14 | 0.15 |
|  |  |  |  |  |  |  |  |  |  |  |  |  |
| *DID Indicator* | 0.76* | 0.19 | 0.36+ | 0.47 | 0.78** | 1.03** | 0.79** | 0.63** | 0.41+ | 0.73* | 0.96** | 0.3 |
|  | 0.35 | 0.27 | 0.21 | 0.33 | 0.23 | 0.24 | 0.24 | 0.24 | 0.23 | 0.31 | 0.16 | 0.21 |
|  |  |  |  |  |  |  |  |  |  |  |  |  |
| Age of Mother | 0.01+ | 0.02* | 0.02* | 0.02* | 0 | 0.02 | 0.02* | 0.01 | 0.02+ | -0.01 | 0 | -0.01 |
|  | 0.01 | 0.01 | 0.01 | 0.01 | 0.01 | 0.01 | 0.01 | 0.01 | 0.01 | 0.01 | 0.01 | 0.01 |
|  |  |  |  |  |  |  |  |  |  |  |  |  |
| *Sex (Male=1)* | | | | | | | | | | | | |
| Female | -0.16 | 0.15 | 0.15+ | 0.02 | -0.04 | -0.03 | 0.01 | -0.05 | -0.14 | -0.11 | -0.04 | 0.02 |
|  | 0.12 | 0.09 | 0.08 | 0.08 | 0.1 | 0.11 | 0.09 | 0.07 | 0.1 | 0.14 | 0.1 | 0.05 |
|  |  |  |  |  |  |  |  |  |  |  |  |  |
| *Locality (Rural=1)* | | | | | | | | | | | | |
| Urban | -0.1 | -0.08 | -0.11 | -0.1 | 0.31* | 0.15+ | 0.03 | 0 | -0.06 | 0.54** | 0.28** | 0 |
|  | 0.11 | 0.13 | 0.07 | 0.08 | 0.13 | 0.09 | 0.06 | 0.09 | 0.12 | 0.2 | 0.1 | 0.07 |
|  |  |  |  |  |  |  |  |  |  |  |  |  |
| Age of Child | 0 | 0.01** | 0.02** | 0.01** | 0 | 0.02** | 0.02** | 0.02** | 0.02** | 0.01** | 0.01** | 0.02** |
|  | 0 | 0 | 0 | 0 | 0 | 0 | 0 | 0 | 0 | 0 | 0 | 0 |
|  |  |  |  |  |  |  |  |  |  |  |  |  |
| *Wealth Quintile (1=1)* | | | | | | | | | | | | |
| 2 | 0.11 | 0.32+ | 0.14 | 0.09 | 0.11 | 0.14 | 0.15 | 0.05 | 0.09 | 0.06 | 0.11 | 0.03 |
|  | 0.08 | 0.18 | 0.15 | 0.08 | 0.1 | 0.21 | 0.21 | 0.09 | 0.13 | 0.28 | 0.17 | 0.08 |
| 3 | 0.15 | 0.27 | 0.27+ | 0.11 | 0.1 | 0.22 | 0.24+ | 0.1 | 0.07 | -0.13 | 0.05 | 0 |
|  | 0.1 | 0.17 | 0.16 | 0.1 | 0.16 | 0.2 | 0.14 | 0.13 | 0.13 | 0.32 | 0.1 | 0.12 |
| 4 | 0.36** | 0.35+ | 0.18 | 0.15 | -0.11 | 0.29 | 0.25+ | 0.25* | 0.15 | -0.05 | 0.15 | 0.07 |
|  | 0.11 | 0.19 | 0.13 | 0.13 | 0.13 | 0.2 | 0.13 | 0.11 | 0.14 | 0.44 | 0.13 | 0.13 |
| 5 | 0.51** | 0.35 | 0.08 | 0.03 | -0.09 | 0.37 | 0.23+ | 0.27* | 0.41 | -0.02 | -0.08 | 0.04 |
|  | 0.12 | 0.31 | 0.19 | 0.13 | 0.26 | 0.24 | 0.14 | 0.12 | 0.26 | 0.56 | 0.15 | 0.1 |
|  |  |  |  |  |  |  |  |  |  |  |  |  |
| *Religion (Hindu=1)* | | | | | | | | | | | | |
| Muslim | -0.06 | 0.05 | -0.19+ | -0.05 | -0.08 | -0.21 | -0.16 | -0.06 | -0.19 | 0.39 | 0.32* | 0.05 |
|  | 0.14 | 0.19 | 0.1 | 0.12 | 0.16 | 0.21 | 0.11 | 0.11 | 0.22 | 0.24 | 0.13 | 0.08 |
| Christian | 0.26 | 0.19 | 0.04 | 0.1 | 0.47** | 0.14 | -0.09 | 0.04 | 0.32 | 0.04 | 0.28 | -0.21 |
|  | 0.25 | 0.21 | 0.15 | 0.18 | 0.18 | 0.26 | 0.14 | 0.14 | 0.24 | 0.2 | 0.18 | 0.2 |
| Sikh | -0.23 | 0.28* | -0.19 | -0.08 | 1.51** | -0.1 | -0.11 | -0.32+ | 0.11 | 1.11** | 0.24 | 0.18 |
|  | 0.19 | 0.12 | 0.35 | 0.18 | 0.34 | 0.34 | 0.15 | 0.17 | 0.21 | 0.39 | 0.23 | 0.26 |
| Other | 0.02 |  | 0.37* | 0.12 | 0.66** | 0.18 | 0 | 0.05 | -0.16 |  | 0.03 | -0.38+ |
|  | 0.18 |  | 0.18 | 0.17 | 0.14 | 0.28 | 0.21 | 0.18 | 0.17 |  | 0.18 | 0.21 |
|  |  |  |  |  |  |  |  |  |  |  |  |  |
| *Caste (General=1)* | | | | | | | | | | | | |
| Scheduled tribe | 0.05 | -0.05 | 0.02 | -0.06 | -0.23 | -0.07 | -0.13 | 0.02 | -0.18 | -0.49+ | 0.03 | 0.04 |
|  | 0.18 | 0.17 | 0.14 | 0.16 | 0.17 | 0.15 | 0.14 | 0.09 | 0.2 | 0.26 | 0.15 | 0.08 |
| Other backward caste | 0.37** | 0.06 | 0.03 | 0.05 | 0.25 | 0.01 | 0.03 | 0.16 | 0.23 | -0.29+ | 0.21+ | 0.09 |
|  | 0.14 | 0.08 | 0.09 | 0.09 | 0.16 | 0.1 | 0.08 | 0.11 | 0.2 | 0.16 | 0.12 | 0.08 |
| Scheduled caste | 0.36** | 0.44** | 0.12 | 0.06 | 0.03 | 0.30+ | 0.13 | 0.20* | 0.06 | -0.09 | 0.09 | 0 |
|  | 0.1 | 0.14 | 0.1 | 0.12 | 0.14 | 0.16 | 0.15 | 0.09 | 0.14 | 0.17 | 0.14 | 0.07 |
|  |  |  |  |  |  |  |  |  |  |  |  |  |
| *No schooling* | | | | | | | | | | | | |
| Primary or lower | 0.22 | 0.22 | 0.20+ | 0.01 | 0.14 | 0.18 | 0.18+ | 0.13 | -0.1 | -0.12 | 0.09 | 0.20* |
|  | 0.15 | 0.22 | 0.11 | 0.11 | 0.14 | 0.14 | 0.1 | 0.11 | 0.22 | 0.2 | 0.14 | 0.1 |
| Middle to Secondary | 0.16 | 0.36 | 0.19 | 0.08 | 0.29+ | 0.21 | 0.18+ | 0.11 | 0 | 0.34 | 0.16 | 0.30** |
|  | 0.13 | 0.25 | 0.15 | 0.11 | 0.17 | 0.18 | 0.11 | 0.09 | 0.19 | 0.28 | 0.14 | 0.08 |
| Graduate | 0.1 | 0.63+ | 0.31 | 0.17 | 0.2 | 0.45* | 0.24 | 0.08 | -0.12 | 0.44 | 0.19 | 0.41** |
|  | 0.15 | 0.32 | 0.21 | 0.14 | 0.24 | 0.19 | 0.15 | 0.1 | 0.19 | 0.47 | 0.19 | 0.15 |
|  |  |  |  |  |  |  |  |  |  |  |  |  |
| *Household size (<5=1)* | |  |  |  |  |  |  |  |  |  |  |  |
| > 5 | 0.05 | -0.13 | -0.15+ | 0.02 | -0.03 | -0.04 | 0.15* | 0.19* | 0.1 | -0.22 | 0.02 | 0.07 |
|  | 0.1 | 0.09 | 0.08 | 0.08 | 0.12 | 0.1 | 0.06 | 0.08 | 0.11 | 0.18 | 0.08 | 0.06 |
|  |  |  |  |  |  |  |  |  |  |  |  |  |
| *Institutional Delivery (Institutional=1)* | | | | | | | | | | | | |
| Non-Institutional | -0.44** | -0.18+ | -0.32** | -0.26** | -0.74** | -0.16 | -0.19+ | -0.25* | -0.54** | -0.46** | -0.47** | -0.28** |
|  | 0.1 | 0.1 | 0.12 | 0.09 | 0.11 | 0.13 | 0.11 | 0.1 | 0.14 | 0.13 | 0.11 | 0.09 |
|  |  |  |  |  |  |  |  |  |  |  |  |  |
| *Distance to Vaccination Site (< 15 minutes=1)* | | | | | | | | | | | | |
| 15 to 30 minutes | -0.15+ | -0.16 | -0.11 | -0.09+ | 0.04 | 0.08 | 0 | -0.05 | 0 | -0.01 | -0.03 | -0.12 |
|  | 0.09 | 0.11 | 0.07 | 0.05 | 0.14 | 0.11 | 0.1 | 0.06 | 0.11 | 0.21 | 0.13 | 0.08 |
| > 30 minutes | -0.41** | -0.38** | -0.38** | -0.24** | -0.16 | -0.27+ | -0.35** | -0.36** | -0.27+ | 0.17 | -0.15 | -0.15 |
|  | 0.15 | 0.09 | 0.11 | 0.09 | 0.15 | 0.16 | 0.1 | 0.11 | 0.16 | 0.25 | 0.17 | 0.1 |
|  |  |  |  |  |  |  |  |  |  |  |  |  |
| Observations | 3689 | 7210 | 7048 | 6925 | 7333 | 6845 | 7230 | 7221 | 4645 | 6375 | 7905 | 7259 |
| Pseudo R^2^ | 0.124 | 0.163 | 0.165 | 0.137 | 0.228 | 0.211 | 0.244 | 0.199 | 0.161 | 0.236 | 0.198 | 0.172 |

*Note: Full=* 1 dose BCG and measles, 3 doses of DPT and polio*; HepB0*=Hepatitis B given at birth, *DPT*=Diphtheria, Pertussis, Tetanus, *BCG*=Bacillus Calmette–Guérin; *OPV=Oral Polio Vaccine; OPV0=OPV* birth dose*;*  *Measles1*= First dose of measles; *OTV*= On-time vaccination – considers timely vaccination of DPT and full immunization.; +p-value<0.10, *p-value<0.05, **p-value<0.01; Standard errors below coefficients.

**Figure S1: Timeline of MI and INCHIS phases**

#
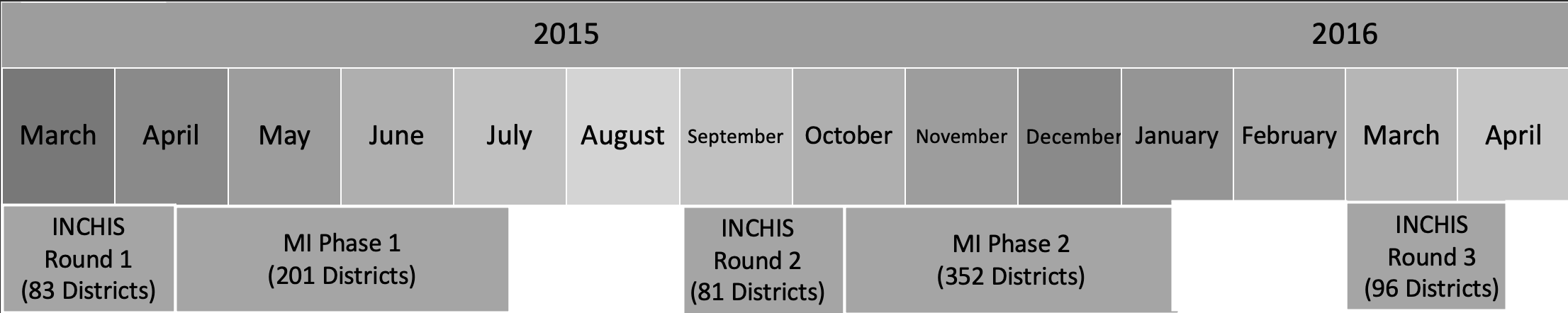


# Note: *INCHIS* = Integrated Child Health and Immunization Survey.

# Figure S2: Full Immunization Interaction Effects


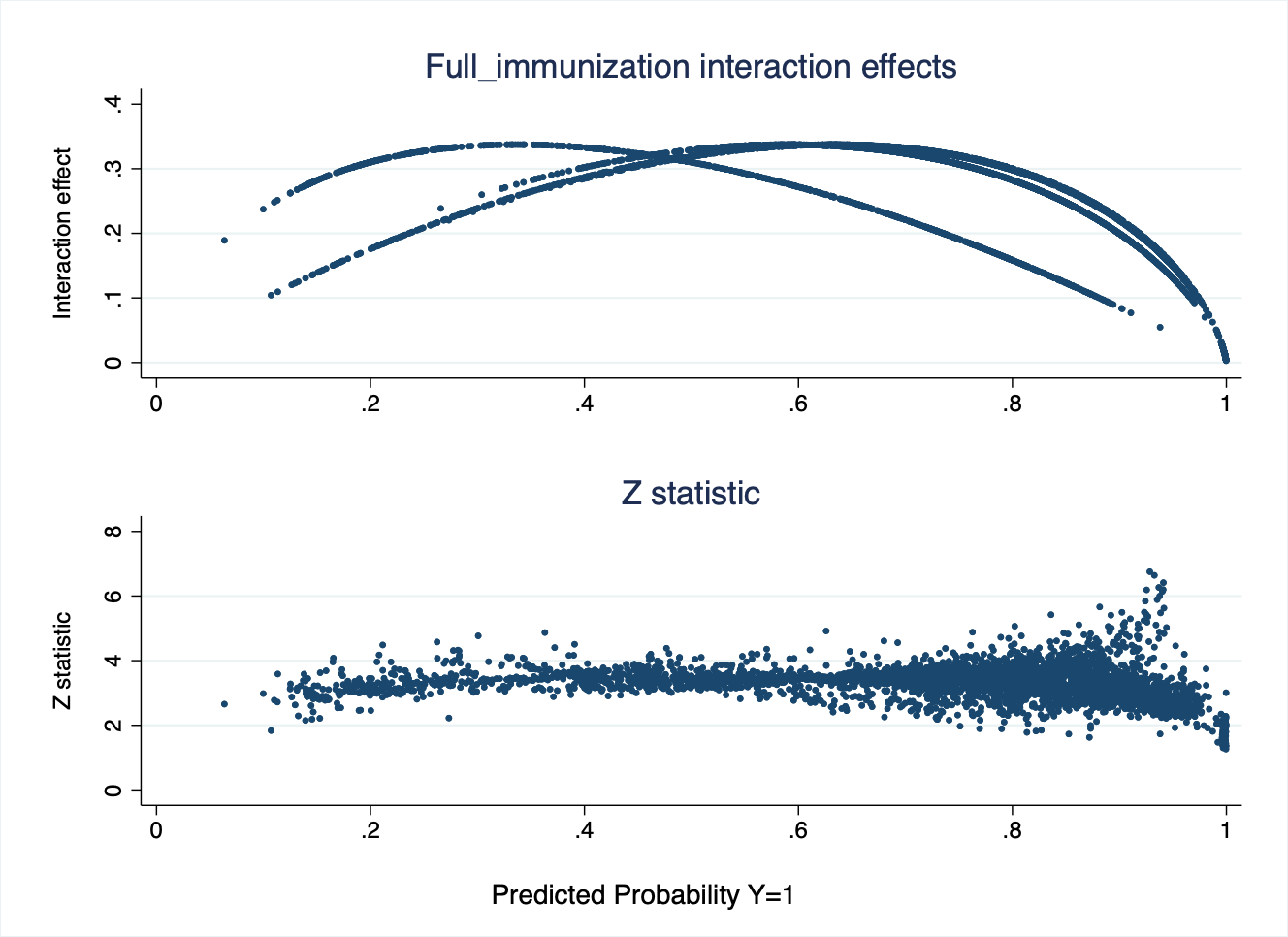

*Note:* Interaction effects and significance levels from probit regression of receipt of vaccine on difference-in-difference indicators and vector of household control variables; includes district level fixed effects. *Full=* 1 dose BCG and measles, 3 doses of DPT and polio*; HepB0*=Hepatitis B given at birth, *DPT*=Diphtheria, Pertussis, Tetanus, *BCG*=Bacillus Calmette–Guérin.

Figure S3: DPT1 Interaction Effects
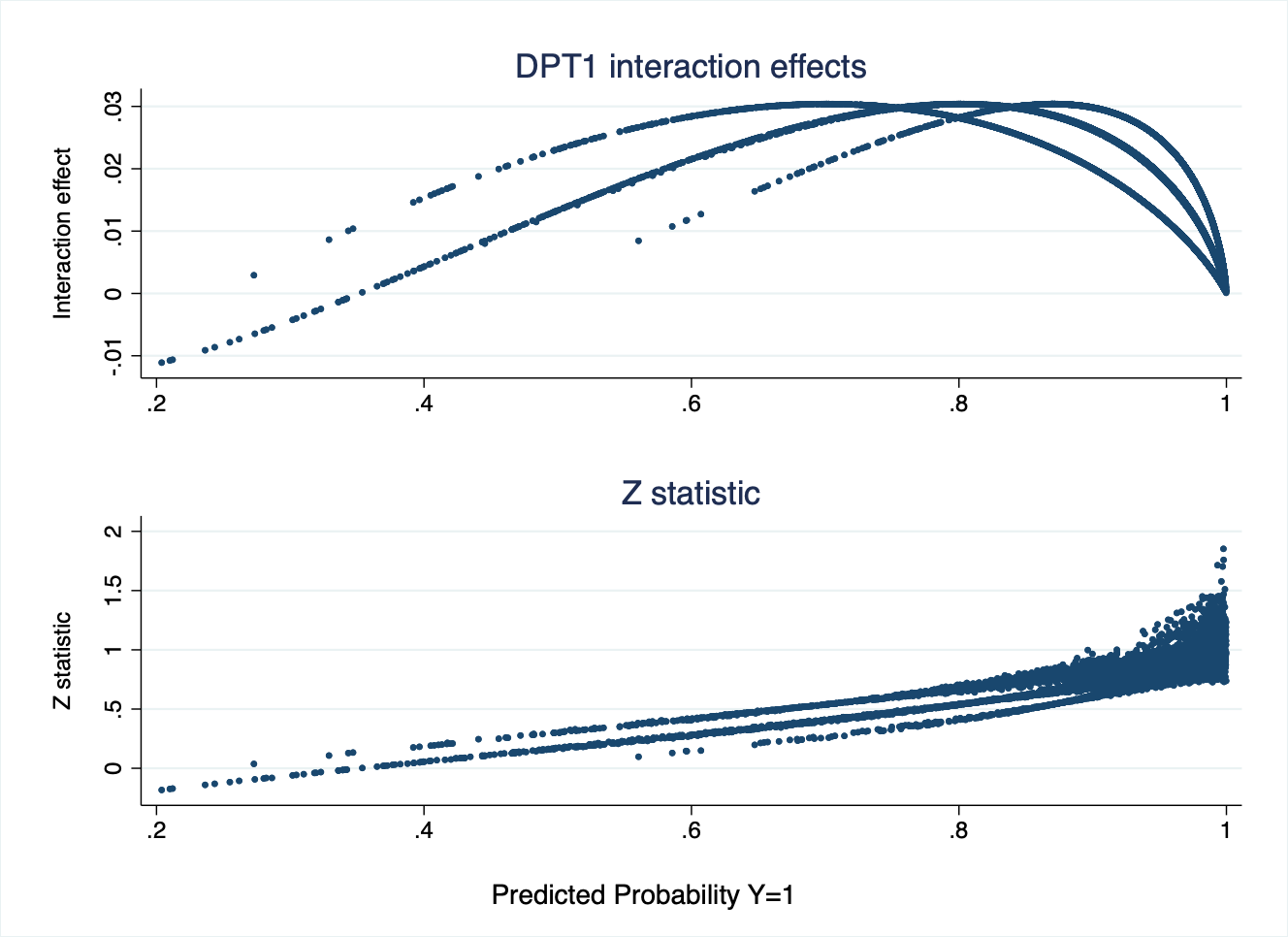


*Note:* Interaction effects and significance levels from probit regression of receipt of vaccine on difference-in-difference indicators and vector of household control variables; includes district level fixed effects. *DPT1*=Diphtheria, Pertussis, Tetanus, dose 1.

Figure S4: DPT2 Interaction Effects
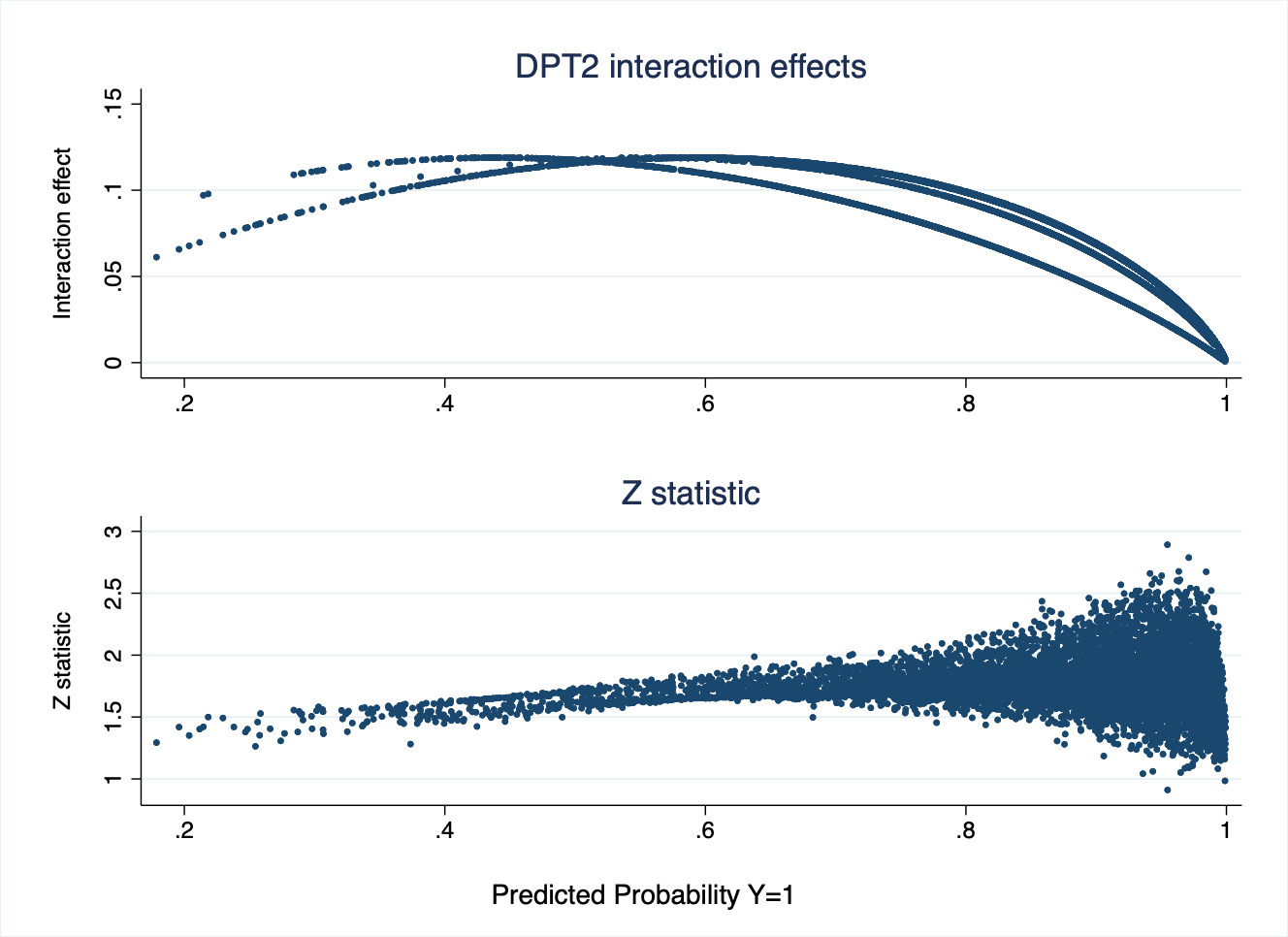

*Note:* Interaction effects and significance levels from probit regression of receipt of vaccine on difference-in-difference indicators and vector of household control variables; includes district level fixed effects. *DPT2*=Diphtheria, Pertussis, Tetanus, dose 2.

# Figure S5: DPT3 Interaction Effects


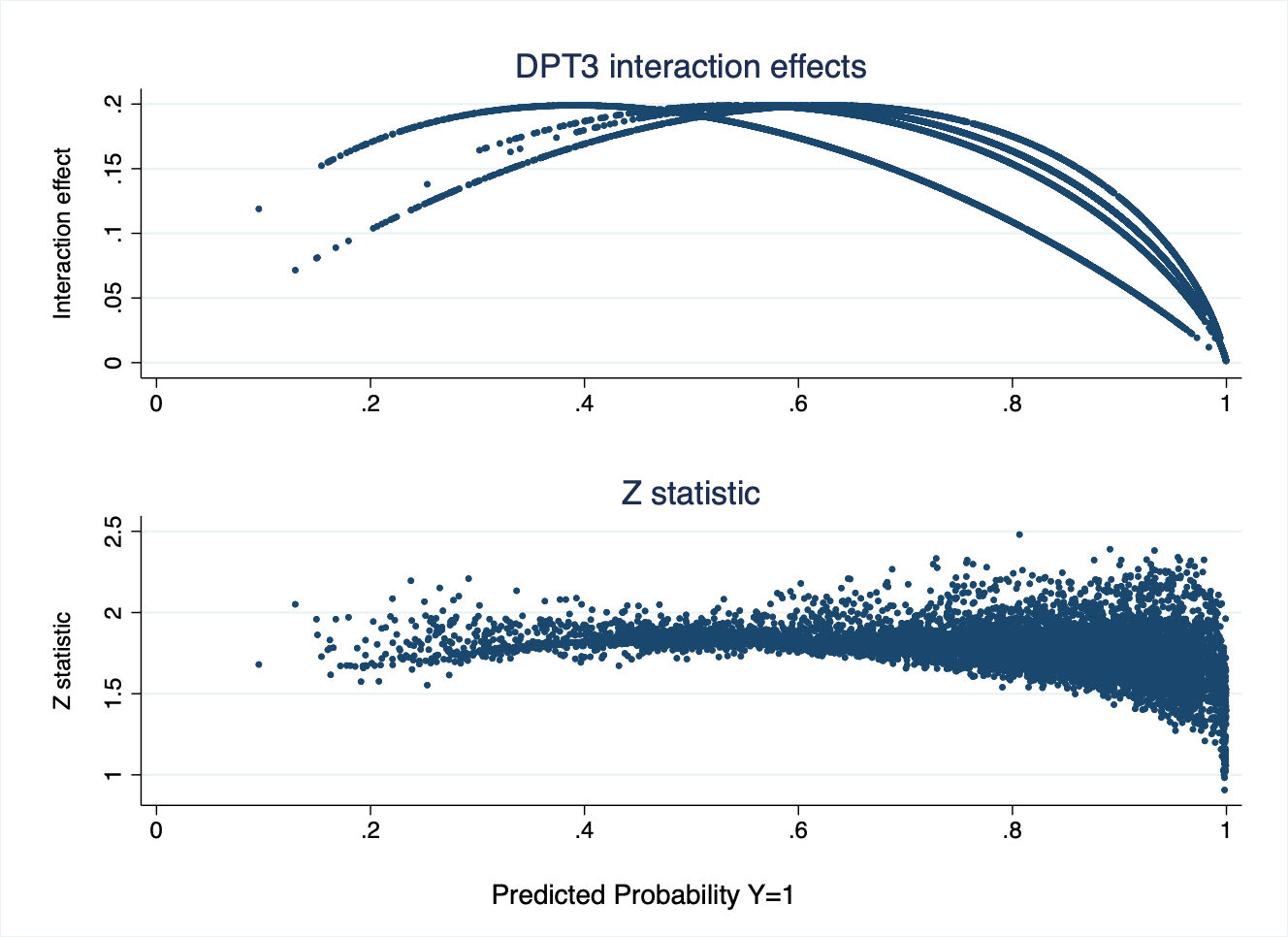


*Note:* Interaction effects and significance levels from probit regression of receipt of vaccine on difference-in-difference indicators and vector of household control variables; includes district level fixed effects. *DPT3*=Diphtheria, Pertussis, Tetanus, dose 3.

Figure S6: OPV0 Interaction Effects

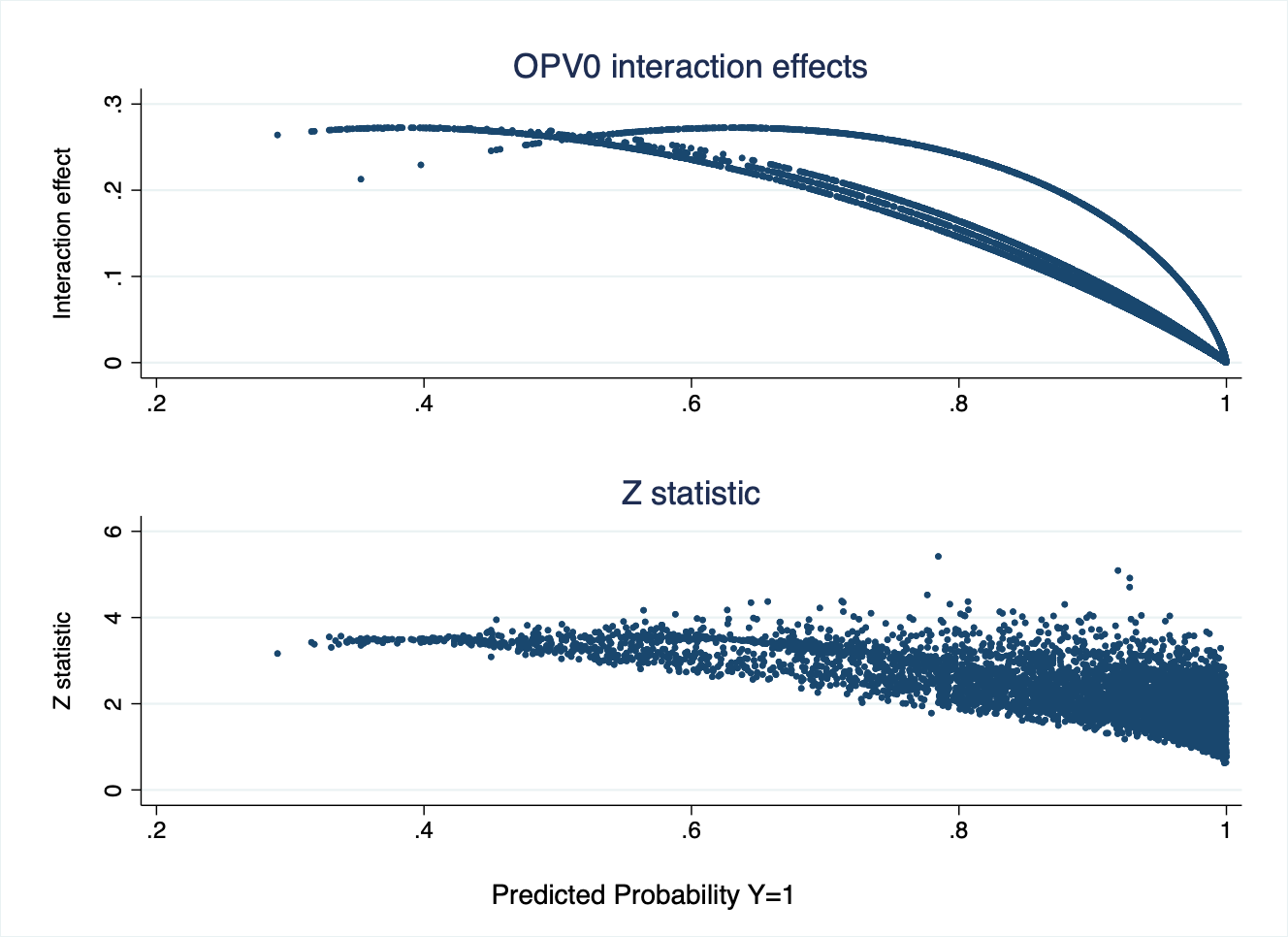

*Note:* Interaction effects and significance levels from probit regression of receipt of vaccine on difference-in-difference indicators and vector of household control variables; includes district level fixed effects. *OPV0=*Oral Polio Vaccine, birth dose.

Figure S7: OPV1 Interaction Effects

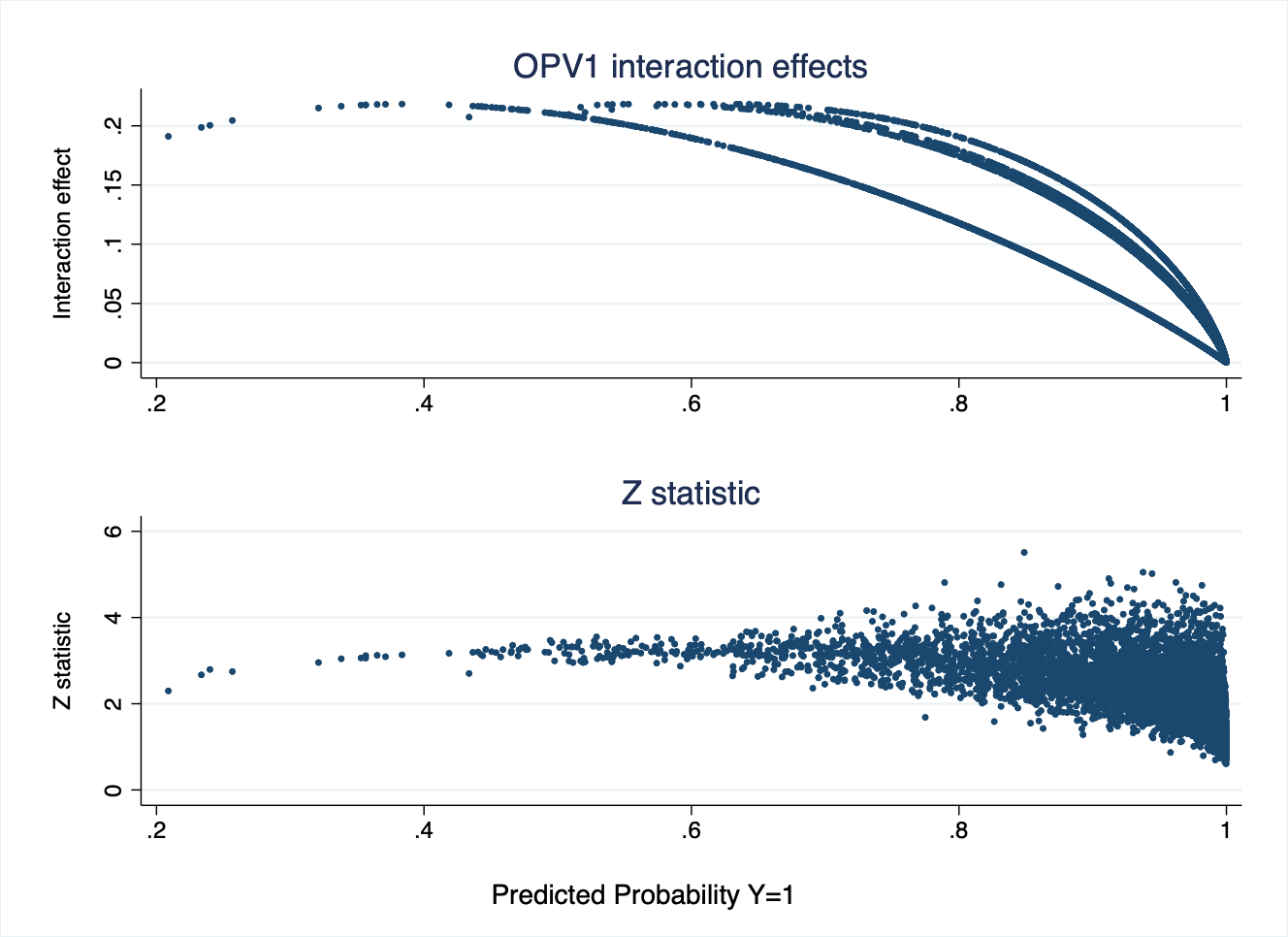

*Note:* Interaction effects and significance levels from probit regression of receipt of vaccine on difference-in-difference indicators and vector of household control variables; includes district level fixed effects. *OPV1=*Oral Polio Vaccine, dose 1.

Figure S8: OPV2 Interaction Effects

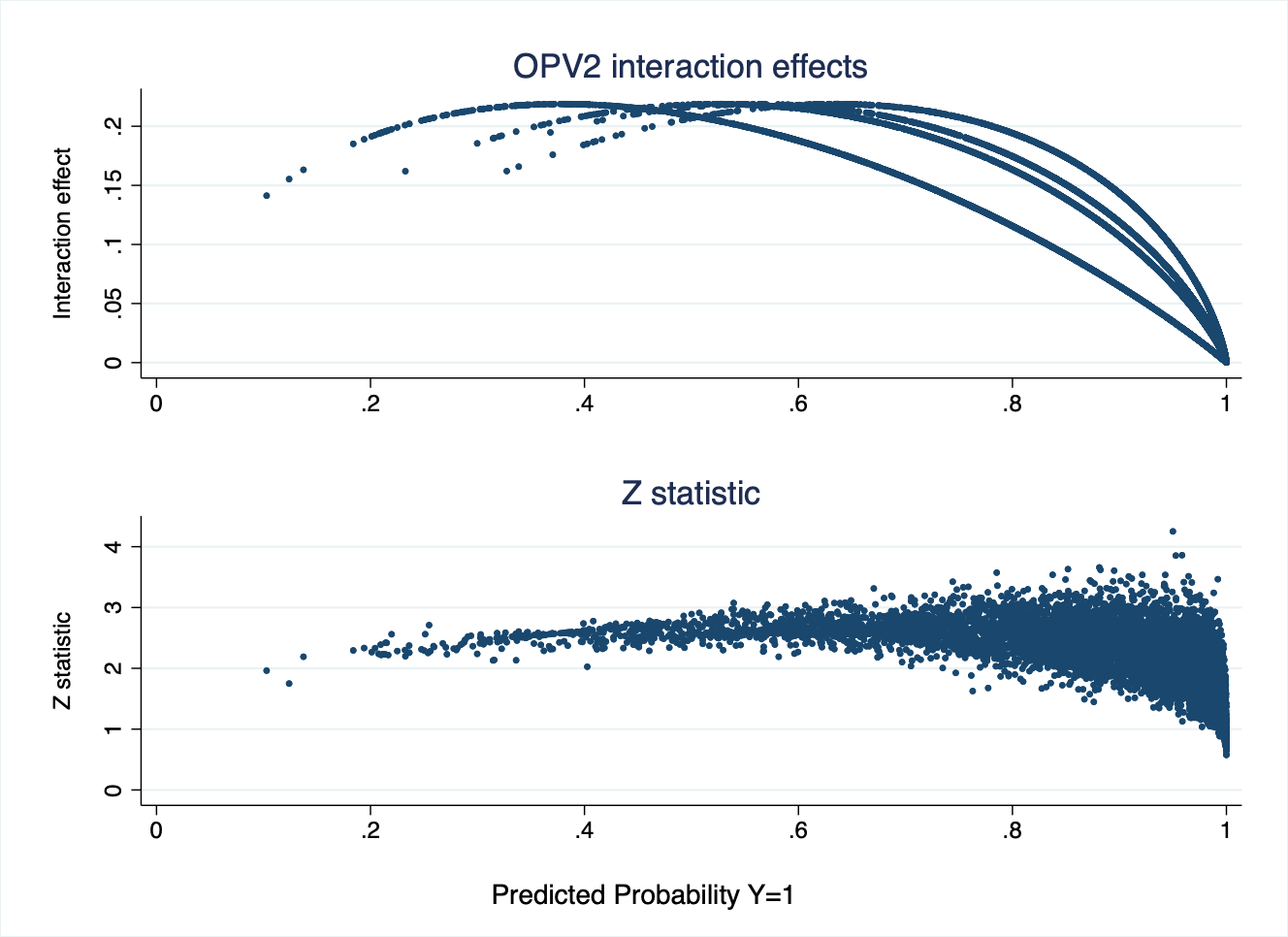

*Note:* Interaction effects and significance levels from probit regression of receipt of vaccine on difference-in-difference indicators and vector of household control variables; includes district level fixed effects. *OPV2=*Oral Polio Vaccine, dose 2.

Figure S9: OPV3 Interaction Effects
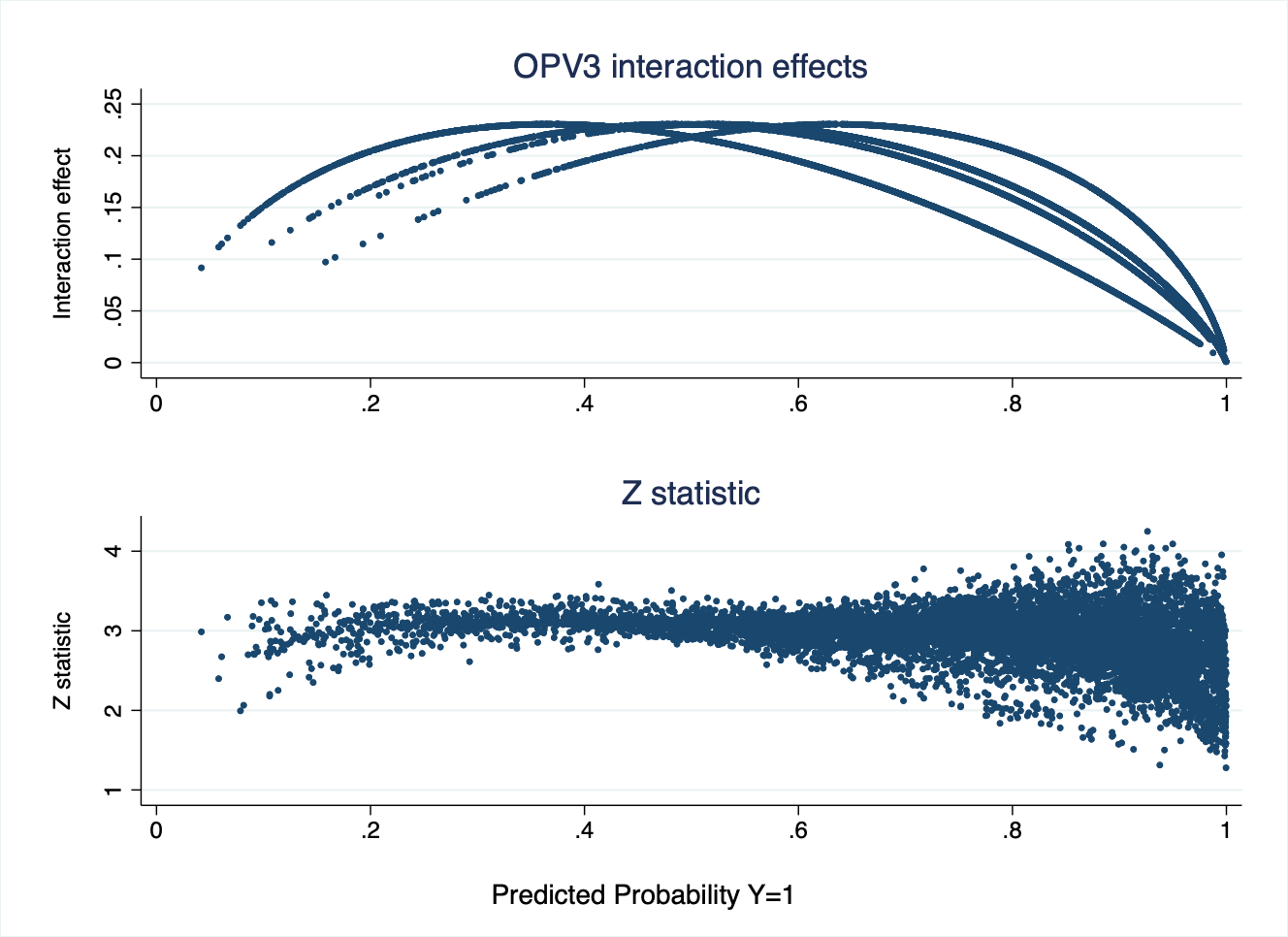

*Note:* Interaction effects and significance levels from probit regression of receipt of vaccine on difference-in-difference indicators and vector of household control variables; includes district level fixed effects. *OPV3=*Oral Polio Vaccine, dose 3.

# Figure S10: Measles Interaction Effects
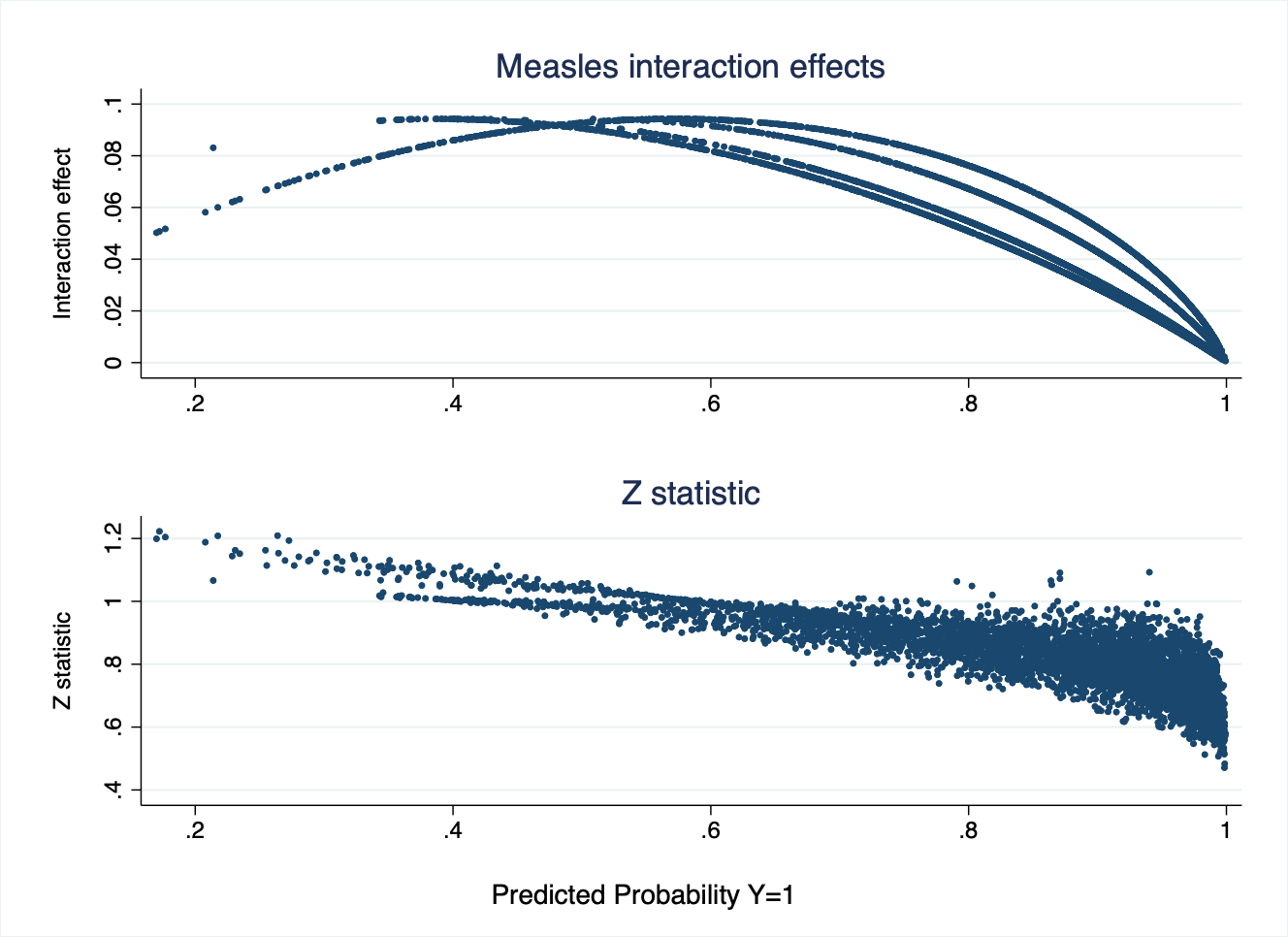


*Note:* Interaction effects and significance levels from probit regression of receipt of vaccine on difference-in-difference indicators and vector of household control variables; includes district level fixed effects.

# Figure S11: BCG Interaction Effects

#
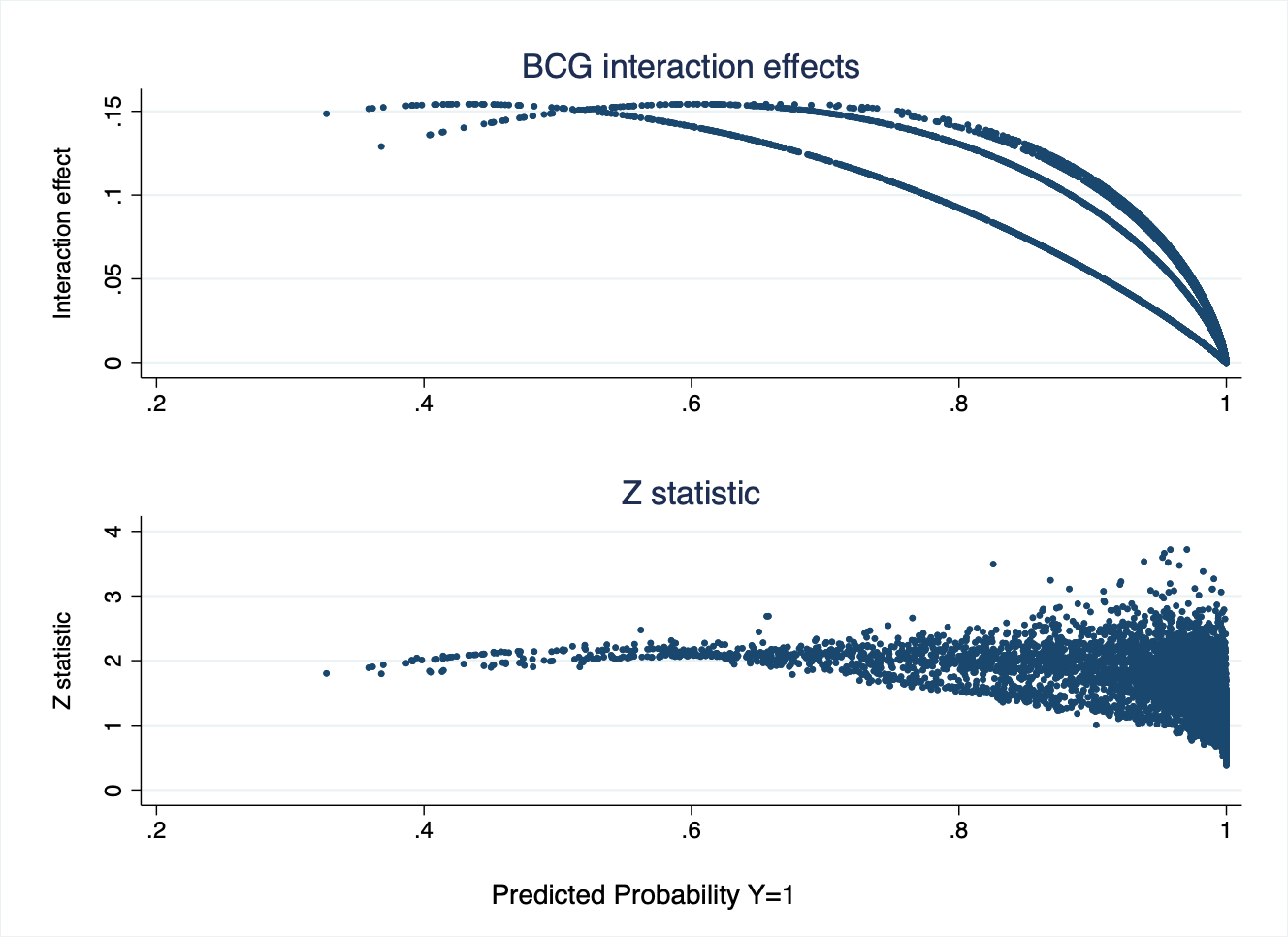


*Note:* Interaction effects and significance levels from probit regression of receipt of vaccine on difference-in-difference indicators and vector of household control variables; includes district level fixed effects. *BCG*=Bacillus Calmette–Guérin.

Figure S12: Hepatitis B Birth Dose Interaction Effects


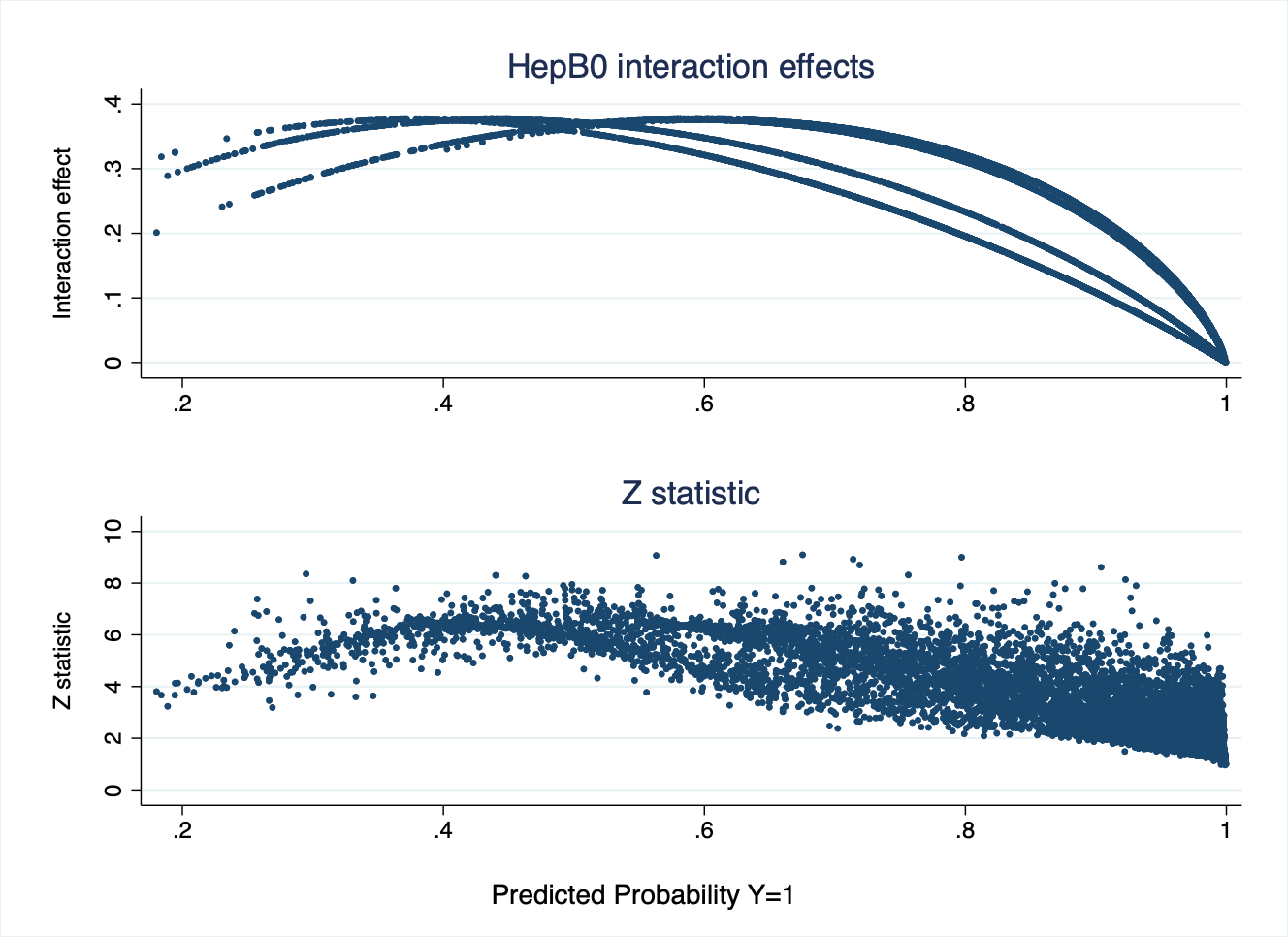


*Note:* Interaction effects and significance levels from probit regression of receipt of vaccine on difference-in-difference indicators and vector of household control variables; includes district level fixed effects.

Figure S13: OTV Interaction Effects


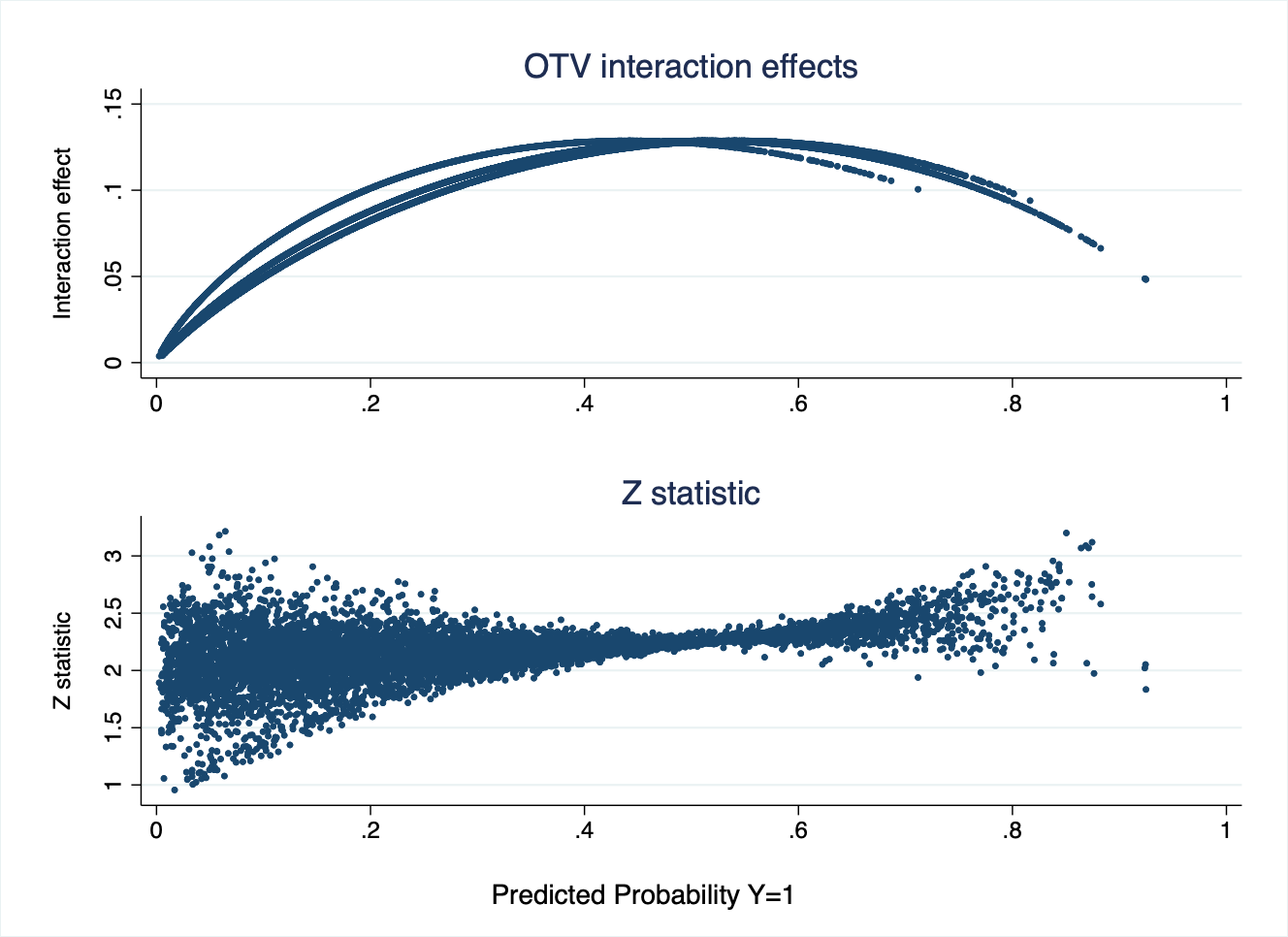


*Note:* Interaction effects and significance levels from probit regression of receipt of vaccine on difference-in-difference indicators and vector of household control variables; includes district level fixed effects. *OTV=* on-time vaccination.

**References**

1. Filmer, D. & Pritchett, L. H. Estimating Wealth Effects without Expenditure Data-or Tears: An Application to Educational Enrollments in States of India. *Demography* **38**, 115–132 (2001).

2. Wold, S., Esbensen, K. & Geladi, P. Principal component analysis. *Chemometrics and Intelligent Laboratory Systems* **2**, 37–52 (1987).

3. Vashishtha, V. M. *et al.* Indian Academy of Pediatrics (IAP) recommended immunization schedule for children aged 0 through 18 years--India, 2014 and updates on immunization. *Indian pediatrics* **51**, 785–800 (2014).
